# UGT2B15 Acts as a Critical Detoxification Barrier Against Chemi-Cal-Induced Hepatotoxicity and Carcinogenesis via the Androgen Receptor Axis

**DOI:** 10.3390/cells15090824

**Published:** 2026-04-30

**Authors:** Yiru Zhao, Yin Wang, Yu Li, Shuqiang Liu, Zhen Jia, Ying Wang, Rong Zhang, Zhongqiu Liu, Linlin Lu

**Affiliations:** 1 Guangdong Provincial Key Laboratory of Translational Chinese Medicine, Joint International Research Laboratory of Translational Cancer Research of Chinese Medicines, International Institute for Translational Chinese Medicine, School of Pharmaceutical Sciences, Guangzhou University of Chinese Medicine, Guangzhou 510006, Chinazhangrong@gzucm.edu.cn (R.Z.); 2 State Key Laboratory of Traditional Chinese Medicine Syndrome, State Key Laboratory of Dampness Syndrome of Chinese Medicine, Guangzhou University of Chinese Medicine, Guangzhou 510006, China; 3 Chinese Medicine Guangdong Laboratory (Hengqin Laboratory), Hengqin 519000, China; 4 School of Pharmaceutical Sciences, Shandong First Medical University (Shandong Academy of Medical Sciences), Jinan 250117, China

**Keywords:** targeted sequencing, UGTs, hepatocellular carcinoma, carcinogen, liver injury

## Abstract

**Highlights:**

**What are the main findings?**

- UGT2B15 is the most frequently mutated UGT gene (44.74%) in Chinese hepatocellular carcinoma patients, with mutations primarily impairing enzymatic activity rather than protein stability.
- UGT2B15 expression is transcriptionally regulated by the androgen receptor (AR), establishing an AR–UGT2B15 detoxification axis in the liver.
- UGT2B15 loss exacerbates CCl_4_- and ethanol-induced hepatotoxicity by dysregulating carcinogen-metabolizing genes including ADH1C, CYP1B1, MUC1, and PTGS2.

**What are the implications of the main findings?**

- High UGT2B15 expression correlates with improved overall survival in HCC patients, suggesting its potential as a prognostic biomarker.
- UGT2B15 acts as a critical chemopreventive barrier against chemical-induced liver injury, identifying it as a potential target for preventive interventions in carcinogen-exposed populations.

**Abstract:**

Uridine diphosphate glucuronosyltransferases (UGTs) are critical phase II detoxification enzymes; however, their mutational landscape and protective roles against chemical carcinogenesis in hepatocellular carcinoma (HCC) remain poorly defined. Here, targeted sequencing of ten liver-enriched UGT genes in 38 paired tissues from a Chinese HCC cohort revealed striking mutation frequencies in UGT2B15 (44.74%), UGT2B10 (36.84%), and UGT2B17 (26.32%). This genomic instability was accompanied by a profound downregulation of UGT2B15 mRNA (9.02-fold decrease, *p* < 0.001) and protein levels (Z-score = 2.32, *p* = 0.0093) in tumors, with higher UGT2B15 expression correlating with improved overall survival in TCGA cohorts (HR = 1.724, *p* = 0.012). Mechanistically, we identified the androgen receptor (AR) as a direct transcriptional regulator of UGT2B15 and UGT2B17, with dihydrotestosterone (DHT) inducing dose-dependent increases in their expression, thereby linking endocrine signaling to hepatic detoxification. Transcriptomic profiling following UGT2B15 knockdown in HCC cells revealed a significant enrichment in chemical carcinogenesis-related pathways. Crucially, UGT2B15 deficiency severely exacerbated carbon tetrachloride (CCl_4_)- and ethanol-induced hepatotoxicity both in vitro and in vivo. Our study uncovers a profound impairment of UGT-mediated detoxification in HCC and establishes the AR–UGT2B15 axis as a critical barrier against chemical-induced liver injury, highlighting its potential as a chemopreventive target in carcinogen-exposed populations.

## 1. Introduction

Hepatocellular carcinoma (HCC) is a leading cause of cancer-related mortality worldwide, with approximately 80% of cases occurring in sub-Saharan Africa and East Asia. According to the International Agency for Research on Cancer (IARC), there were over 840,000 new cases and 780,000 deaths globally in 2018, with nearly half of these cases reported in China [1,2]. Major risk factors include metabolic liver diseases and exposure to environmental carcinogens such as aflatoxin B1 (AFB1) and aristolochic acid (AA) [3,4]. Mechanistically, AFB1 promotes HCC development by inducing the TP53 R249S mutation [5], while AA metabolites form DNA adducts that drive characteristic A > T transversions in genes such as TP53 and JAK1 [6]. Accumulating evidence suggests that dysregulation of hepatic detoxification systems, particularly drug-metabolizing enzymes, plays a crucial role in carcinogen-induced hepatocarcinogenesis [7].

Cytochrome P450 (CYP) enzymes, highly expressed in the liver and intestine, are involved in both phase I detoxification and reactive oxygen species (ROS) generation, with excessive ROS contributing to oxidative stress that accelerates HCC progression [8,9]. UDP-glucuronosyltransferases (UGTs) are another major class of metabolizing enzymes, widely expressed in human cancers and frequently mutated across tumor types [10,11]. Polymorphisms in UGT genes (e.g., UGT2B4 in breast cancer and UGT1A6 in endometrial cancer) have been associated with cancer risk [12,13]. Despite the liver being the central organ for xenobiotic metabolism and detoxification, where UGTs are most abundantly expressed, the mutational landscape and functional significance of UGTs in HCC, particularly in high-risk Chinese populations, remain largely unexplored.

The UGT superfamily in mammals comprises four families (UGT1, UGT2, UGT3, and UGT8), with UGT1 and UGT2 playing predominant roles in drug metabolism and cancer susceptibility [14]. Although some studies link UGT1A7*3 alleles to HCC risk [15,16,17] and have demonstrated an association between reduced metabolic capacity and increased risk of liver cancer [18,19], others suggest a protective role for low-activity UGT variants in certain populations [20], highlighting ethnic and molecular heterogeneity. A pan-cancer analysis revealed frequent somatic UGT mutations [11], yet population-specific profiles in high-incidence regions like China remain lacking. Given the high morbidity and mortality of HCC in China, systematically delineating UGT mutational patterns in this population is clinically imperative.

UGT2B15, which catalyzes the glucuronidation of steroid hormones and is critically involved in hormone inactivation, has been implicated in hormone-dependent and gender-disparate cancers such as prostate and breast cancer [21,22]. The pronounced sexual dimorphism in HCC incidence implicates sex hormone signaling, with the androgen receptor (AR) promoting and estrogen receptor (ER) suppressing hepatocarcinogenesis [23]. Although higher UGT2B15 activity has been observed in male liver [24], its expression, regulation, and functional role in HCC remain undefined. Beyond glucuronidation, UGT isoforms such as UGT2B7 can influence cellular metabolism and proliferation [25], raising the question of whether UGT2B15 exerts non-canonical roles in liver cancer.

Therefore, this study aimed to systematically characterize UGT genetic and expression alterations in Chinese HCC patients and elucidate the functional role of UGT2B15 in hepatocarcinogenesis. We performed integrated targeted sequencing of 19 UGT genes, transcriptomic profiling, and proteomic analysis in tumor and adjacent non-tumor tissues. Our results demonstrated that (1) UGT genes, particularly UGT2B15, harbor recurrent somatic mutations with the highest frequency (44.74%) in the UGT family; (2) UGT2B15 is significantly downregulated in HCC tumors at both mRNA and protein levels and is transcriptionally regulated by AR signaling; and (3) UGT2B15 loss impairs the cellular response to xenobiotic-induced damage by dysregulating the expression of phase I/II carcinogen-metabolizing enzymes, including ADH1C and CYP1B1, and inflammation-related genes such as PTGS2 and MUC1. These findings provide the first comprehensive characterization of UGT alterations in Chinese HCC and identify UGT2B15 as a potential tumor suppressor that links androgen-regulated hormone metabolism to carcinogen detoxification.

## 2. Materials and Methods

### 2.1. Cell Lines and Reagents

The Huh7 and MHCC-97H HCC cell lines (ATCC, RKV, USA) authenticated by STR analysis were cultured in DMEM medium (10% FBS) and incubated at 37 °C in 5% CO_2_. QIAamp DNA Mini Kit was bought from QIAGEN (Hilden, Germany). Ion AmpliSeq™ Library Kit 2.0, Ion Xpress Barcode Adapters 1–16, Ion Library Quantitation Kit, Ion PGM (TM) Hi-Q (TM) OT2 Kit, Ion 318™ Chip Kit v2 BC, and ION PGM^TM^ Hi-Q^TM^ Sequencing Kit were purchased from Life Technologies (Grand Island, NY, USA); 5-dihydrotestosterone (DHT), 4-hydroxy-flutamide (Flut), sorafenib, paracetamol, S-oxazepam, S-oxazepam-G, vorinostat, and vorinostat-G were purchased from Melone Pharmaceutical Co., Ltd. (Dalian, China). Primary antibodies against AR, UGT2B15, and GAPDH, along with the corresponding secondary antibodies, were obtained from Abcam, Inc. (Abcam, CA, USA). All UGT signature peptides were synthesized by APeptide Co., Ltd. (Shanghai, China). The shUGT2B15 Plasmid LV5 (EF-1aF/GFP&Puro) and over-expressed UGT2B15 plasmid LV5 (EF-1aF/GFP&Puro) were bought from GenePharma Co., Ltd. (Shanghai, China).

### 2.2. Patients and Tissue Specimens

Subjects were obtained from 38 Chinese HCC consecutive patients who underwent surgical resection between June 2011 and June 2015 at the Nanfang Hospital (Guangzhou, Canada). Approvals for tissue collection and studies were obtained from the Nan Fang Hospital Research Ethics Committee. Informed consent was obtained from each patient in accordance with the Declaration of Helsinki. All specimens were confirmed by pathological examination and clinicopathological parameters. Samples were obtained from viable tumor and non-tumor liver tissue located > 2 cm away from the primary lesion. Tumors with evidence of prior therapy, such as radiofrequency ablation or trans-arterial chemo-embolization were excluded. Detailed clinical information for these patients is listed in Supplementary Table S1.

### 2.3. DNA Extraction and Sequencing Libraries

DNA was isolated from fresh-frozen tissue samples using the QIAamp DNA MiniKit (Qiagen) following manufacturer’s instructions. Sequencing was performed on 38 tumor–normal paired samples using the Ion Torrent PGM with the Ion PGM TM 200 Sequencing Kit (Ion Torrent, Life Technologies, Shanghai, China) containing 318 chips. We obtained an average read length of 102 bp and 23.6 Mb of raw reads per specimen, with a depth of 1076x covering 94.75% of amplicons. Detailed procedures are described in the Supplementary Materials.

Binary sequence alignment map files (BAM files) have been deposited in the National Center for Biotechnology Information dbGAP database (SRA 529312).

### 2.4. Activity Determination of UGT2B15 and UGT2B17

S-oxazepam and vorinostat were used as corresponding substrates for UGT2B15 and UGT2B17, respectively. The glucuronidation activities were determined in tumor liver microsomes (HLMs) and the adjacent normal HLMs collected from 10 HBV-positive HCC patients, and the pooled commercial normal human liver HLMs were used as a reference. Equal amounts of microsomal protein (0.265 mg/mL) from different tissues were used to determine the glucuronidation enzymatic activities of individual UGTs.

### 2.5. Plasmid Construction and Transient Cell Transfection

The designed short hairpin RNA (shRNA) against the UGT2B15 construct contained a unique 21-nt double-stranded UGT2B15 sequence that presented as an inverted complementary repeat, a loop sequence (5′-CTCGAG-3′), an RNA PloIII terminator (5′-TTTTTT-3′), and 5′ single-stranded overhangs for ligation into the AgeI- and EcoRI-digested LV10-U6-RFP&Puro lentivirus vector (GenePharma, Shanghai, China). The negative control vector (pGLV–NC–shRNA) contained a nonsense shRNA insert in order to control any effects caused by nonRNAi mechanisms. The validation of UGT2B15 knockdown was performed by qRT-PCR and immunoblotting.

### 2.6. Gene Expression Microarray of UGT2B15 Knockdown in MHCC-97H Cells

MHCC-97H cells were harvested 72 h after transfection with shUGT2B15 or shControl. Approximately 1 × 10^6^ cells from each sample were subjected to gene microarray assay. Total RNA was extracted using TRIzol reagent (Invitrogen, Carlsbad, CA, USA) following the manufacturer’s instructions and analyzed with Agilent SurePrint G3 Human GE 8 × 60 K Microarray (Agilent technologies). Gene expression fold changes were compared between shUGT2B15- and shControl-treated cells and genes with FC > 2 were considered differentially expressed. Microarray data were deposited in the NCBI GEO database (https://www.ncbi.nlm.nih.gov/geo/subs/) (accessed on 12 February 2019) with accession number GSE97387.

### 2.7. PDX Establishment

All animal studies were approved by the Animal Care and Use Committee (IACUC) at Guangzhou University of Chinese Medicine. The experimental animals were maintained in a specific pathogen-free animal room at a temperature of 25 ± 1 °C, relative humidity of 65 ± 10%, and a 12/12 h light/dark cycle (lights on at 8:00 am). In compliance with the protocol approved by the Nan Fang Hospital Research Ethics Committee and with the subject’s informed consent, a piece of surgically resected tumor tissue was used for xenotransplantation. Briefly, patient samples were collected, trimmed, sliced into 20–30 mm^3^ fragments and implanted subcutaneously in the fore and/or hind bilateral flanks of anesthetized 6- to 8-week NOD/SCID Il2rg^−/−^ (NSI) mice (Shanghai SLAC Laboratory Animal Co., Ltd.; Shanghai Sino-British Sippr/BK Lab Animal Co., Ltd., Shanghai, China) within three hours. The mice were examined periodically for three months. Tumor size was measured using a digital caliper (Cal Pro, Sylvac, Yverdon-les-Bains, Switzerland) and calculated using the following formula: 0.5 × length × width^2^.

### 2.8. Statistical Analyses

Data were represented as the mean ± standard deviation (SD) of at least three independent experiments and plotted by GraphPad Prism software (GraphPad Software 6.0, San Diego, CA, USA). Data were statistically analyzed using SPSS 18.0. * *p* < 0.05, ** *p* < 0.01 and *** *p* < 0.001 were considered statistically significant.

Allele imbalance was analyzed using an online method (http://gvs.gs.washington.edu/GVS144/GenotypeFileInput, accessed on 5 February 2017) as previously reported.

## 3. Results

### 3.1. UGT2B15 Exhibits the Highest Mutation Frequency Among UGT Family Members in Chinese HCC Patients

**Figure 1:**
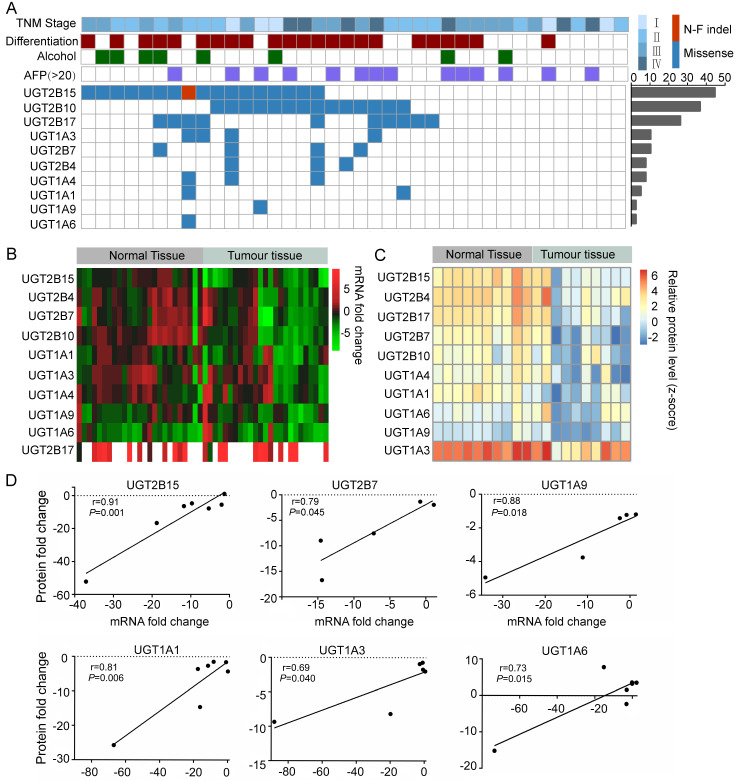
**Quantitative portrait of ten UGTs in HCC patients.** (**A**) Overview of mutations and major associated clinical features. The heatmap shows genes (rows) and tumors (columns) with missense mutations (blue) or non-frameshift indel (red). The major clinical, pathological and molecular features of each tumor are shown as boxes: tumor lymph node metastasis (blue), poor differentiation (dark red), alcohol exposures (green) and alpha fetoprotein level (violet). (**B**) qRT-PCR analysis of gene expression profiling of ten UGTs in HCC tissue (*n* = 25). The heatmap was generated by log transformation of real-time PCR data presented as 2^−△△Ct^. (**C**) UGT protein expression in 10 HCC tumors and adjacent normal tissue via LC-MS/MS. Protein expression levels of 10 UGT isozymes in human liver microsomes prepared from tumor tissues and adjacent normal tissue of 25 HCC patients. The heatmap was generated by log transformation of values detected by LC-MS/MS (pM/mg). (**D**) Pairwise correlation of UGTs between protein reduction ratios and mRNA fold changes.

To investigate the somatic mutation spectrum of liver-specific UGT genes in hepatocellular carcinoma (HCC), we performed targeted sequencing on paired tumor-adjacent tissue samples from 38 Chinese HCC patients, covering 10 UGT genes (5 UGT1A and 5 UGT2B subfamily members). A total of 100 somatic mutations were identified, comprising 99 missense mutations and 1 insertion/deletion mutation. Among these, eight mutations (8%) were predicted to be deleterious by SIFT algorithm (Figure 1A). Comparison with OMIM and COSMIC databases revealed that 8 mutations had been previously documented, while 91 were novel SNPs. Genes with mutation frequencies >5% included UGT2B15 (44.74%, 17/38 patients), UGT2B10 (36.84%, 14/38), UGT2B17 (26.32%), UGT1A3 (10.53%), UGT2B7 (10.53%), UGT2B4 (7.89%), UGT1A4 (7.89%), and UGT1A1 (5.26%), with UGT2B15 showing the highest mutation rate among all UGT isoenzymes analyzed.

### 3.2. The Gene, Protein and Activity of UGTs Were Reduced in HCC Tumors

mRNA and protein expression of 10 UGT isoenzymes were analyzed via qRT-PCR and label-free LC-MS/MS. Results showed that except for UGT2B17, which was undetectable in 13 samples, the remaining 9 UGT isoenzymes were detectable in both HCC tumors and adjacent non-cancerous tissues (Figure 1B). Compared with adjacent non-cancerous tissue, the majority of UGT genes exhibited significant downregulation in tumor tissue ranging from 2.0-fold to 52.2-fold, with UGT2B10, UGT2B15, and UGT2B7 showing the most pronounced downregulation reductions. Conversely, UGT2B17 demonstrated an 8-fold upregulation (Figure 1B). Protein-level analysis revealed that UGT proteins were generally lower in tumor microsomes than in adjacent non-cancerous tissue (Figure 1C). UGT1A3 and UGT2B4 exhibited the highest basal expression levels, both of which were significantly reduced in tumors. UGT2B15 (Zmax = 2.32, *p* = 0.0093) and UGT2B17 (Zmax = 2.11, *p* = 0.012) exhibited the most pronounced differential expression. Correlation analysis revealed a significant positive correlation between the downregulation ratio of seven UGT proteins (including UGT2B15) and their mRNA downregulation ratio (Figure 1D), whereas no significant correlation was observed for UGT2B17. These findings indicate widespread suppression of UGT expression and function in HCC, suggesting a potential regulatory role in HCC development.

### 3.3. The Expression of UGT2B15 Was Associated with Highly Mutations and Survival Outcome

**Figure 2:**
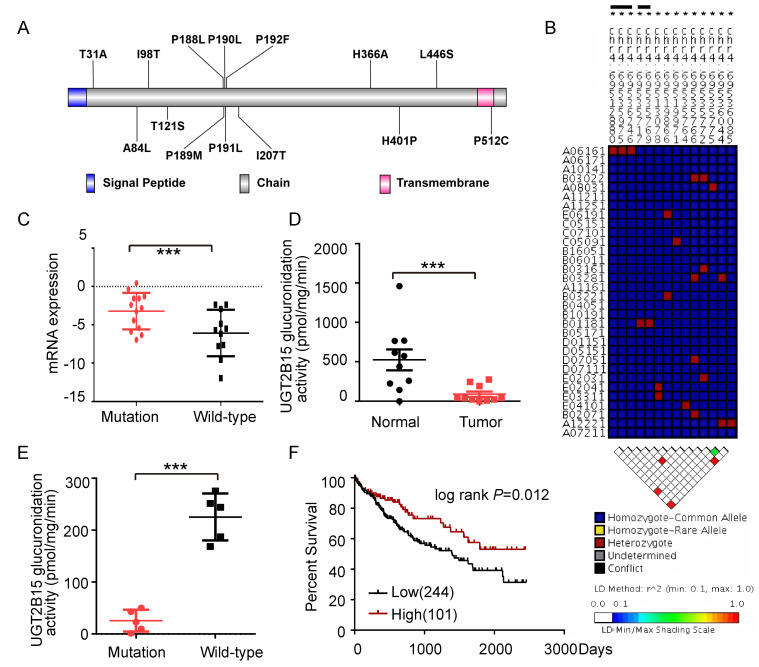
**The genomic, transcription, translation and enzymatic activities and good prognosis of UGT2B15 in HCC.** (**A**) Mutation distribution in UGT2B15 functional domains. Blue, signal peptide; red, transmembrane region; gray, main protein chain. (**B**) Visual genotype and linkage disequilibrium (LD) plot of UGT2B15. Each column indicates one SNP, while each array denotes one individual. Blue and red represent the homozygous common allele and heterozygous genotype, respectively. The LD plot is based on pairwise γ^2^ values. (**C**) Differential mRNA expression of UGT2B15 gene between mutant and wild-type cases. (**D**) Enzymatic glucuronidation activity of UGT2B15 in human liver microsomes prepared from tumor tissues and adjacent normal tissue of 10 HCC patients. Representative data from three independent experiments are shown as the mean ± S.D. (**E**) Enzymatic glucuronidation activity of UGT2B15 in mutated and wild-type human liver microsomes prepared from tumor tissues. * *p* < 0.05, ** *p* < 0.01, and *** *p* < 0.001. (**F**) Kaplan–Meier curves of the cumulative survival of HCC patients with low and high UGT2B15 mRNA expression using the TCGA dataset. (*n* = 345, HR = 1.724 (1.118, 2.393), *p* < 0.05).

Given that UGT2B15 exhibited both the highest mutation frequency and the most significant expression reduction, we focused on investigating its role in HCC. Sequencing results revealed that UGT2B15 was mutated in 17 out of 38 HCC patients (44.74%), with a total of 15 distinct mutations identified. Ten of these mutations were located in the substrate-binding domain, and three were in the UDPGA-binding domain, suggesting potential impairment of its enzymatic function (Figure 2A). Allele frequency analysis revealed linkage disequilibrium between multiple coding region mutations and tag SNPs (Figure 2B). Interestingly, mRNA expression of UGT2B15 was significantly higher in mutant tumors than in wild-type tumors (*p* = 0.017; Figure 2C), while its protein levels showed an opposite trend (*p* = 0.094). Importantly, enzyme activity was significantly lower in mutant tumors than in wild-type tumors (*p* < 0.0001; Figure 2D,E), demonstrating that mutations primarily impair catalytic function rather than protein stability. Enzyme activity was highly correlated with protein expression (R^2^ = 0.84, *p* < 0.001). Importantly, TCGA data analysis revealed that high UGT2B15 expression was significantly associated with prolonged overall survival in patients (HR = 1.72, *p* = 0.012; Figure 2F). The favorable prognosis associated with high UGT2B15 expression reflects the function of the wild-type protein, whereas UGT2B15 mutations lead to loss of detoxification function and promote HCC progression. These findings suggest that UGT2B15 mutations may influence HCC clinical prognosis by altering its expression and function.

### 3.4. Androgen Regulated UGT2B15 and UGT2B17 in HCC

**Figure 3:**
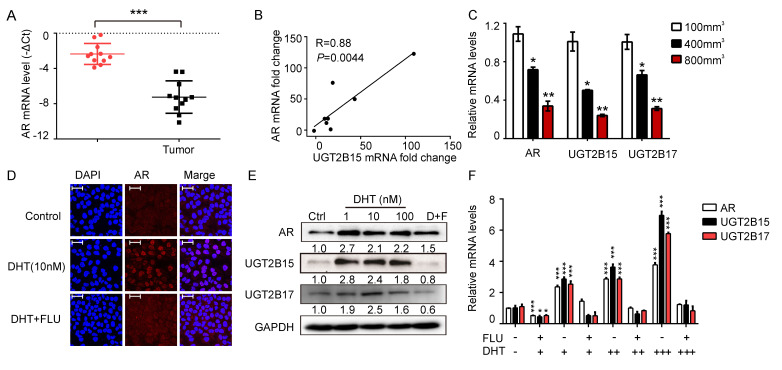
**Androgen receptor signaling controls the transcription of UGT2B15 and UGT2B17 in HCC.** (**A**) AR gene expression in tumor tissues and corresponding adjacent normal tissues (*n* = 38). (**B**) Pearson correlation analysis of mRNA expression between AR and UGT2B15 in tumor tissues (*n* = 38). (**C**) qRT-PCR analysis of AR, UGT2B15 and UGT2B17 at different tumor sizes in patient-derived xenograft HCC model. GAPDH was used as the internal control. Data are represented as mean ± SD (*n* = 3). * *p* < 0.05, ** *p* < 0.01. (**D**) Representative immunofluorescence images of AR in MHCC-97H cells treated with indicated concentration of DHT for 48 h as measured by confocal microscopy. Scale bar: 25 μm. (**E**) Western blot of UGT2B15 and UGT2B17 induced by DHT in MHCC-97H cells. GAPDH is presented as the equivalent loading controls. (**F**) mRNA levels of AR, UGT2B15, and UGT2B17. MHCC-97H cells treated with 1 nM DHT (+), 10 nM DHT (++), 100 nM DHT (+++), FLU (100 nM) or vehicle (0.1% DMSO) for 24 h. GAPDH was used for normalization. Representative data from three independent experiments are shown as the mean ± S.D. * *p* < 0.05, ** *p* < 0.01, and *** *p* < 0.001.

We hypothesize that despite the high mutation frequency of UGT2B15 in tumors, its gene and protein expression show a downregulated trend, suggesting upstream transcriptional regulation. Given that the androgen receptor (AR) has been reported to regulate UGT2B15 and UGT2B17 in prostate and breast cancers, we investigated whether this regulatory axis operates in HCC. Analysis of clinical samples revealed significantly downregulated AR expression in HCC tumor tissues compared to adjacent non-cancerous tissues (Figure 3A). AR mRNA expression showed a strong positive correlation with UGT2B15 mRNA expression (R^2^ = 0.88, *p* < 0.01; Figure 3B). In PDX models, expression of AR, UGT2B15, and UGT2B17 decreased synchronously as tumors grew (Figure 3C). To establish causality, we treated MHCC-97H cells with DHT which induced a significant dose-dependent upregulation of AR, UGT2B15, and UGT2B17 at both the transcriptional and protein levels (*p* < 0.001, Figure 3D–F), while the AR antagonist flutamide completely abolished this effect (Figure 3E,F). In summary, our results demonstrate that AR is a direct transcriptional regulator of UGT2B15 and UGT2B17 in HCC cells.

### 3.5. UGT2B15 Has No Influence on Cellular Functions in HCC Cells

**Figure 4:**
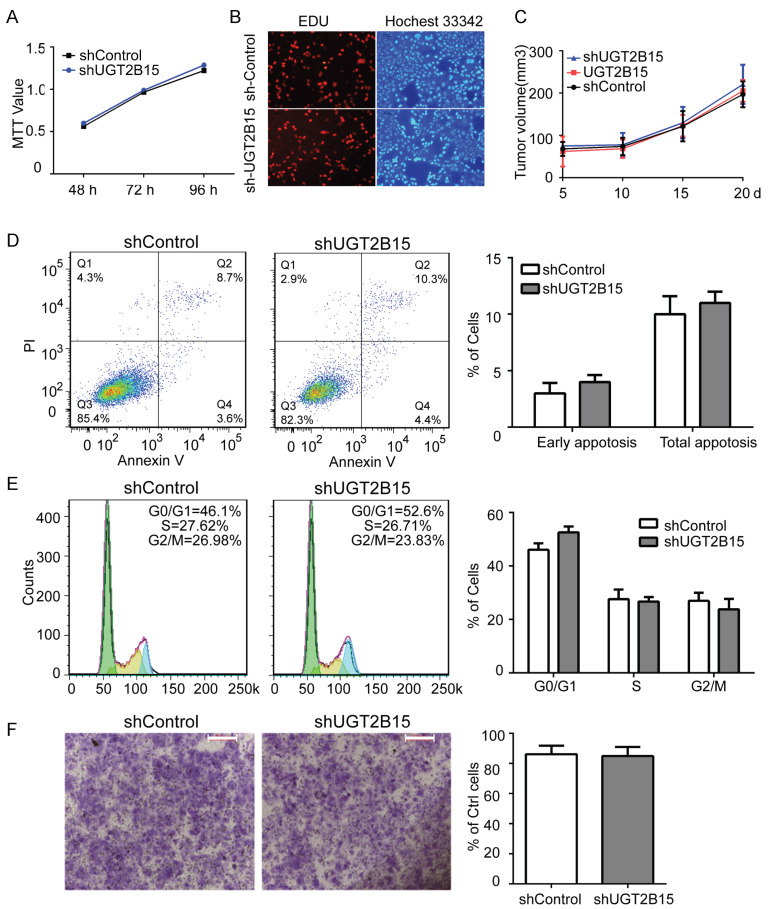
**UGT2B15 silencing has no influence on liver cell function.** (**A**) Cell viability of Huh7 cells transduced with shControl and shUGT2B15 as assessed by MTT assay. (**B**) Cell proliferation of Huh7 cells with shControl and shUGT2B15 labeled with EdU (red) and Hoechst33342 (blue). Scale bar: 50 μm. (**C**) Tumor volumes of xenograft mice treated with shControl, shUGT2B15 or UGT2B15 over 20 days. (**D**) Cell cycle analysis with PI staining was performed on Huh7 cells after UGT2B15-silencing by flow cytometry. (**E**) Flow cytometry analysis of apoptotic cells after UGT2B15 silencing with annexin-V/PI staining. (**F**) Invasive ability of Huh7 cells treated with shControl and shUGT2B15 was assessed by Matrigel invasion assay. Scale bar: 200 μm.

Given the significantly reduced expression and loss of function of UGT2B15 in tumors, we investigated its impact on canonical oncogenic phenotypes by establishing a stable HCC cell model with its knockdown. Compared to immortalized normal hepatocytes, Huh-7 cells exhibited higher UGT2B15 mRNA levels, prompting the use of shRNA for effective knockdown (89.1% efficiency). Cell viability and EdU proliferation assays revealed that UGT2B15 knockdown had no significant impact on Huh-7 cell survival or proliferation (Figure 4A,B). In vivo mouse xenograft experiments further demonstrated no significant difference in tumor growth between the knockdown group and the control group (Figure 4C). No statistically significant changes were observed in apoptosis and cell cycle distribution (Figure 4D,E). Transwell migration assays showed that UGT2B15 knockdown did not affect cell migration ability (Figure 4F). Collectively, UGT2B15 does not directly regulate the proliferation and invasion of HCC cells.

### 3.6. UGT2B15 Knockdown Altered Gene Expression Profiles in MHCC-97H Cells

**Figure 5:**
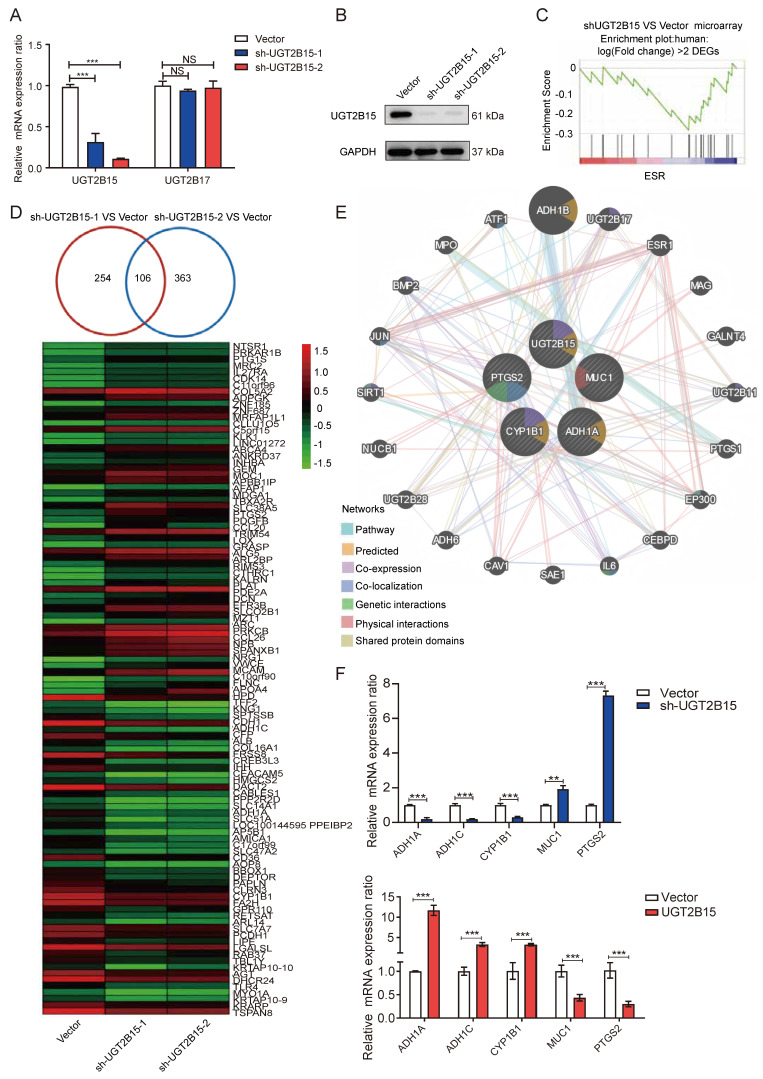
**Differentially expressed genes after UGT2B15 gene knockdown in MHCC-97H cells.** (**A**,**B**) Western blotting analysis and qRT-PCR were used to confirm reduced UGT2B15 expression without affecting UGT2B17 in MHCC-97H cells transfected with vector control or shRNA#1 and shRNA#2 targeting UGT2B15. (**C**) GSEA enrichment of DEGs between shUGT2B15 and shControl. (**D**) Venn diagram depicting the overlap of DEGs identified by each shRNA in MHCC-97H cells. Top 130 differentially expressed genes are shown as heatmap. (**E**) Network analysis of chemical carcinogenesis metabolism pathway performed using GeneMANIA. (**F**) qRT-PCR validated the functionally interacting genes with UGT2B15 following UGT2B15 knockdown or overexpression in MHCC-97H cells. NS (not significant) *p* > 0.05, * *p* < 0.05, ** *p* < 0.01, and *** *p* < 0.001.

To investigate the potential molecular mechanisms of UGT2B15 in HCC, we performed gene expression profiling in MHCC-97H cells stably knocked down for UGT2B15. Compared with control cells, the two shRNAs identified 254 and 363 differentially expressed genes (DEGs), respectively, with 106 genes overlapping (Figure 5D). KEGG pathway enrichment analysis revealed that DEGs were significantly enriched in xenobiotic metabolism pathways, including metabolism of flavones, ketone bodies, butyrate, and chemical carcinogens, as well as multiple signaling pathways including TNF, cAMP, and HIF-1 (Supplementary Table S5). GSEA analysis indicated a shift of DEGs toward ESR1 target genes (Figure 5C). Further integration with GeneMANIA network analysis (Figure 5E) led to the selection of five genes related to carcinogen metabolism (ADH1A, ADH1C, CYP1B1, MUC1, and PTGS2) for validation. qRT-PCR results showed that ADH1A, ADH1C, and CYP1B1 expression was significantly downregulated (3.40–5.29-fold) upon UGT2B15 knockdown, while MUC1 and PTGS2 were markedly upregulated (Figure 5F); overexpression of UGT2B15 exhibited the opposite trend. These findings indicate that UGT2B15 modulates the response of HCC cells to chemical carcinogen-induced damage by regulating the expression of genes associated with chem xenobiotic metabolism.

### 3.7. UGT2B15 Protected Against Chemical Carcinogenesis-Induced Liver Injury In Vitro and In Vivo

**Figure 6:**
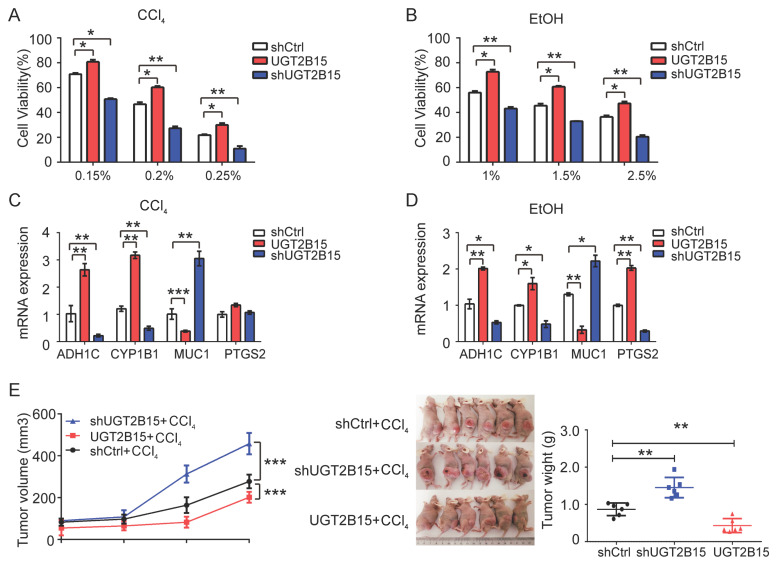
**Hepatoprotective effects of UGT2B15 against chemical-induced liver injury.** (**A**,**B**) Cell viability of MHCC-97H cells expressing shControl, UGT2B15 or shUGT2B15 in the presence of CCl_4_ (**A**) or EtOH (**B**) over 72 h. Each value represents the mean ± SD of three independent experiments. * *p* < 0.05, ** *p* < 0.01, and *** *p* < 0.001. Cell viability of MHCC-97H cells expressing shControl or UGT2B15 or shUGT2B15 in the presence of CCl_4_ (**C**) or EtOH (**D**) for qRT-PCR analysis of five significantly differentially expressed genes related to chemical carcinogenesis metabolism in MHCC-97 cells transduced with shUGT2B15, shControl or UGT2B15. GAPDH was used for normalization. Representative data from three independent experiments are shown as the mean ± S.D, * *p* < 0.05, ** *p* < 0.01, and *** *p* < 0.001. (**E**) Balb/c-nude mice were subcutaneously injected with MHCC-97 cells stably transfected with shUGT2B15, shControl, or overexpressed UGT2B15 (4 × 10^6^, *n* = 6 each group), followed by exposure to CCl_4_ (0.2%) once tumors were established (diameter < 2 mm) for 20 consecutive days. The tumor growth curve of xenograft mice was recorded and is shown in the left panel (*n* = 6). Representative images of developed tumors and tumor weights at 4 weeks in nude mice are displayed in the middle and right panels, respectively.

We further investigated the role of UGT2B15 in chemically induced liver injury. In vitro experiments revealed that MHCC-97H cells overexpressing UGT2B15 exhibited significant resistance to CCl_4_- and EtOH-induced cytotoxicity (Figure 6A,B), whereas UGT2B15 knockdown exacerbated the damage caused by both toxins. Mechanistically, CCl_4_ and EtOH treatment differentially altered the expression of metabolism-related genes including ADH1C, CYP1B1, MUC1, and PTGS2. Knocking down UGT2B15 amplified the regulatory effects of CCl_4_/EtOH on ADH1C, CYP1B1, and MUC1, whereas UGT2B15 overexpression reversed these changes (Figure 6C,D). In an in vivo xenograft tumor model, CCl_4_ treatment significantly inhibited tumor growth in UGT2B15-overexpressing tumors (inhibition rate 50.2%, *p* < 0.001), whereas UGT2B15 knockdown increased tumor growth by 67.3% under CCl_4_ exposure (*p* < 0.001) (Figure 6E). Collectively, these results indicate that UGT2B15 plays a crucial protective role in chemical-induced liver injury through its interaction with genes involved in xenobiotic metabolism.

## 4. Conclusions

In conclusion, this study provides the first comprehensive characterization of UGT mutational landscape in Chinese HCC patients, identifying UGT2B15 as the most frequently mutated UGT isoenzyme (44.74%) with marked downregulation at both mRNA and protein levels. Mechanistically, we demonstrate that UGT2B15 is transcriptionally regulated by the androgen receptor (AR), establishing an AR–UGT2B15 axis that links endocrine signaling to hepatic detoxification. Functionally, UGT2B15 does not directly regulate HCC cell proliferation or invasion; instead, it serves as a critical detoxification barrier against chemical-induced hepatotoxicity by modulating the expression of carcinogen-metabolizing genes including ADH1C, CYP1B1, MUC1, and PTGS2. Loss of UGT2B15 function exacerbates CCl_4_- and ethanol-induced liver injury, while its overexpression confers protection. These findings position UGT2B15 as a potential prognostic biomarker and chemopreventive target in HCC, particularly for populations with high environmental carcinogen exposure. Future studies should explore UGT2B15 activators or AR modulators as preventive interventions in high-risk individuals and investigate the role of UGT2B15 in drug-induced liver injury and immunotherapy response.

## 5. Discussion

**Figure 7:**
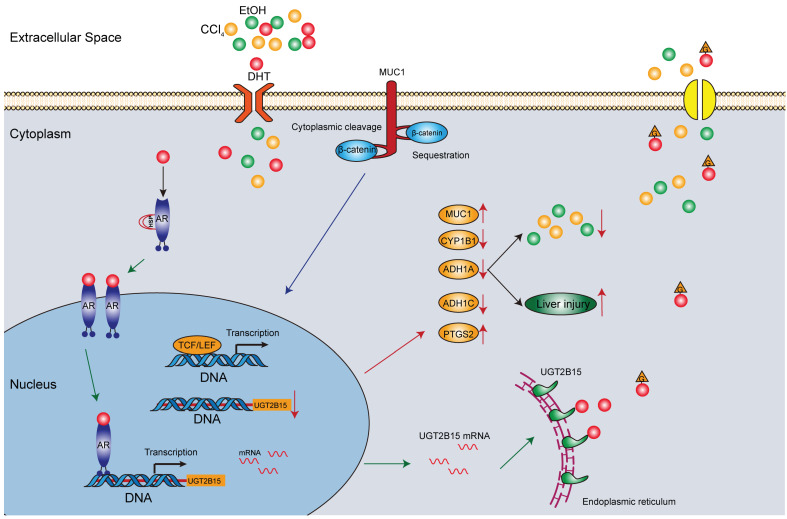
**Schematic model illustrating the protective role of the AR–UGT2B15 axis against chemical-induced hepatotoxicity and carcinogenesis in hepatocellular carcinoma.** Under physiological conditions, androgen receptor (AR) signaling transcriptionally activates UGT2B15 expression, which in turn glucuronidates and detoxifies endogenous steroids and exogenous carcinogens (e.g., CCl_4_ metabolites, aflatoxin B1). UGT2B15 also modulates the expression of downstream genes involved in chemical carcinogenesis, including ADH1C, CYP1B1, MUC1, and PTGS2. In HCC, frequent UGT2B15 mutations (44.74%) and AR downregulation lead to loss of UGT2B15 enzymatic activity and reduced expression, impairing detoxification capacity and exacerbating chemical-induced liver injury, thereby promoting hepatocarcinogenesis.

In this study, we systematically characterized the mutational landscape and functional significance of UDP-glucuronosyltransferases (UGTs) in Chinese hepatocellular carcinoma (HCC) patients. Through integrated targeted sequencing of 10 liver-specific UGT isoenzymes in 38 HCC cases, coupled with transcriptomic, proteomic, and functional analyses, we identified UGT2B15 as the most frequently mutated UGT gene (44.74%) with marked downregulation in tumor tissues (9.02-fold decrease in mRNA; protein Z-score = 2.32; *p* = 0.0093). Mechanistically, we demonstrated that UGT2B15 expression is directly regulated by androgen receptor (AR) signaling, and its high expression correlates with improved overall survival (*p* = 0.012). Importantly, loss- and gain-of-function experiments revealed that UGT2B15 protects against CCl_4_-induced hepatotoxicity by modulating carcinogen-metabolizing genes (ADH1A, ADH1C, CYP1B1, MUC1), providing the first evidence linking UGT2B15 to chemical liver injury prevention. These findings establish UGT2B15 as a potential prognostic biomarker and chemopreventive target in HCC, bridging hormone metabolism with carcinogen detoxification pathways (Figure 7).

Our study reveals a distinct UGT mutational profile in Chinese HCC patients compared to pan-cancer cohorts. Unlike previous pan-cancer UGT mutation studies [11] which reported sporadic mutations across tumor types, we identified high-frequency mutations (>5%) in 8 out of 10 UGT isoenzymes in the Chinese HCC cohort, with concomitant downregulation of UGT gene and protein expression in 80% of tumor tissues (Figure 1B,C). UGT2B15 exhibited the highest mutation frequency (44.74%) among all UGT family members analyzed. We identified 15 distinct mutations across the 17 patients, all of which were different (no recurrent mutation hotspots were observed). The mutations were distributed across functional domains: 10 mutations (66.7%) were located in the substrate-binding domain, and 3 mutations (20%) were in the UDPGA-binding domain. The remaining two mutations were in non-conserved regions. The high mutation frequency of UGT2B15 may be attributed to: (1) its high expression level in hepatocytes, rendering it more susceptible to genotoxic insults from environmental carcinogens such as aflatoxin B1 (common in the Chinese cohort); (2) the presence of mutational hotspots within its exonic regions; and (3) potential selection pressure during hepatocarcinogenesis, as loss of UGT2B15 function may provide a growth advantage under carcinogen exposure. Among these, UGT2B15 exhibited the most pronounced reduction in both gene expression (9.02-fold downregulation) and protein abundance (maximum Z-score Zmax = 2.32; *p* = 0.0093) between tumors and matched normal tissues. Moreover, its high mRNA expression correlated with prolonged overall survival in patients (*p* = 0.012), suggesting UGT2B15 as a potential prognostic biomarker for HCC and providing a basis for molecular subtyping and targeted therapy in Chinese HCC patients. This population-specific mutational enrichment may reflect the unique etiology of HCC in China, where aflatoxin B1 and hepatitis B virus exposure drive distinct carcinogenic pathways [3]. The high prevalence of UGT2B15 mutations (44.74%) in our cohort, which substantially exceeds rates in Western populations, underscores the necessity of ethnicity-tailored genomic profiling for precision oncology.

Our multi-omics analysis uncovered a paradoxical relationship between UGT2B15 mutations and protein expression. We systematically analyzed UGT2B15 at genomic, transcriptomic, proteomic, and functional levels. While genomic (or exonic) mutations are typically thought to promote protein expression or activity, HCC tissues harboring UGT2B15 mutations in our cohort did not exhibit significant reduction in protein levels but demonstrated marked loss of enzyme function, suggesting that mutations primarily impair catalytic activity rather than protein stability. Although genomic, transcriptomic, and proteomic alterations of UGT in the liver have been extensively studied [25,26], our research provides further compelling evidence for AR-mediated transcriptional regulation of UGT2B15 in HCC. We found that the decline in UGT2B15 expression was primarily attributable to AR downregulation rather than other regulators like ESR1 (Figure 3 and Figure 5D), consistent with the known male predominance of HCC and the tumor-suppressive role of androgen signaling in hepatocytes [27]. This finding challenges the conventional view that driver mutations are the primary determinants of gene expression in cancer, highlighting the importance of epigenetic and hormonal contexts in metabolic enzyme regulation.

Furthermore, this study suggests that UGT2B15 may function as a “non-driver gene” in HCC development while playing a crucial role in cellular stress response pathways. Survival analysis supports an association between high UGT2B15 expression and favorable prognosis (Figure 2F), indicating its involvement in HCC pathology. Although UGT2B15 has been implicated in β-adrenergic receptor- or androgen-driven prostate cancer progression [28,29], knocking down UGT2B15 in HCC cells did not significantly affect proliferation or invasive capacity (Figure 4), distinguishing its role in liver versus prostate tumorigenesis. Unexpectedly, gene expression profiling revealed that UGT2B15 influences multiple cellular signaling pathways, including carcinogen metabolism (Figure 5C–E), consistent with prior reports on the interplay between cellular metabolism and glucuronidation signaling [30]. For populations highly exposed to environmental carcinogens (alcohol, aflatoxin B1, etc.), UGT2B15 mutation or low expression can serve as a prognostic biomarker to identify high-risk individuals, supporting early chemoprevention strategies. The AR-UGT2B15 axis represents a potential target for hepatoprotection; activating this pathway may enhance hepatic detoxification and reduce chemical-induced liver injury and carcinogenesis. For HCC patients with low UGT2B15 expression, individualized detoxification-supportive therapy may help improve liver function and tolerance to antitumor treatments. The phenotypic differences between Huh-7 and MHCC-97H cells after UGT2B15 knockdown are indeed closely related to their inherent metabolic and malignant characteristics. MHCC-97H is a highly metastatic HCC cell line with stronger metabolic plasticity and greater dependence on xenobiotic detoxification pathways, whereas Huh-7 cells exhibit a relatively weak detoxification response. Concurrently, we identified novel interactive pathways associated with UGT2B15, suggesting its function may extend beyond classical glucuronidation, with specific mechanisms requiring further elucidation.

The chemopreventive role of UGT2B15 represents a paradigm shift in understanding UGT function in HCC. Subsequent experiments demonstrated that UGT2B15 modulates chemical-induced liver injury by interacting with other metabolic genes. Consistent with prior studies [30,31,32], ethanol is metabolized via the glucuronidation pathway (Figure 6A–D). However, CCl_4_, a known liver injury inducer, had not previously been implicated in UGT-related processes. Our study provides the first experimental evidence that UGT2B15 acts as a chemopreventive factor in carcinogen exposure scenarios. We confirmed in both in vivo and in vitro experiments that UGT2B15 exerts a protective effect against CCl_4_-induced liver injury, while its knockdown exacerbates CCl_4_ hepatotoxicity (Figure 6E). This may result from UGT2B15-mediated glucuronidation and inactivation of CCl_4_ metabolites or the involvement of its downstream target genes in chemically induced carcinogen metabolism. UGT2B15 is an important phase II detoxification enzyme that catalyzes glucuronidation of numerous drugs and herbal components. Therefore, UGT2B15 deficiency is reasonably hypothesized to aggravate drug-induced liver injury (DILI) and herb-induced liver injury (HILI). Knockdown of UGT2B15 significantly disrupted the expression of genes including ADH1A, ADH1C, CYP1B1, MUC1, and PTGS2 (Figure 6C,D). Among these, ADH1A colocalizes with UGT2B15 and is co-expressed with genes such as UGT2B17 and ADH1B [33]; MUC1 is a transmembrane glycoprotein exhibiting oncoprotein functions when glycosylation is abnormal or expression/localization is disrupted [34]; PTGS2 shows inverse expression with UGT2B15 in vitro (Figure 5F). The coordinated downregulation of ADH1A and ADH1C (both encoding alcohol dehydrogenases critical for aldehyde detoxification) upon UGT2B15 loss suggests a transcriptional network linking phase II conjugation with phase I oxidation. Conversely, the reciprocal upregulation of MUC1 and PTGS2, both pro-inflammatory mediators, implies that UGT2B15 suppresses carcinogenic inflammation. Current knowledge regarding the molecular mechanisms underlying UGT2B15 crosstalk with MUC1/PTGS2 is limited, warranting further investigation into their functional associations. Our current study did not directly detect ferroptosis, cuproptosis, or ROS levels. However, transcriptomic analysis showed that UGT2B15 knockdown dysregulates ADH1C, CYP1B1, PTGS2, and other genes associated with oxidative stress and inflammatory responses. CYP1B1 overactivation and PTGS2 upregulation can promote ROS accumulation, which is a core trigger of ferroptosis and chemical hepatotoxicity. Therefore, UGT2B15 deficiency may exacerbate liver injury at least partially through ROS-driven cell death pathways. UGT2B15-mediated detoxification and metabolic homeostasis may shape the tumor immune microenvironment by regulating inflammatory mediators and oxidative stress, thereby potentially affecting the efficacy of immune checkpoint inhibitors (ICIs). However, this hypothesis lacks direct experimental evidence and will be explored in future mechanistic studies.

In summary, this study systematically characterized the somatic mutations, protein/gene expression, and enzymatic activity of UGTs in hepatocellular carcinoma, revealing the pathological association between UGT2B15 and HCC progression, thereby providing novel insights into HCC prognosis. Notably, pharmacologic inhibition experiments highlighted AR’s regulatory role over UGT2B15 and UGT2B17 in the liver, expanding the upstream regulatory network of UGT2B15. Furthermore, UGT2B15′s interactions with multiple cellular pathways extend its potential functions beyond glucuronidation. Most importantly, UGT2B15’s protective effect against liver damage induced by carcinogens like CCl_4_ identifies it as a promising chemopreventive target in populations with high environmental carcinogen exposure, offering a novel strategy for HCC prevention that bridges hormone metabolism with xenobiotic detoxification. Future studies should explore UGT2B15 agonists or AR modulators as preventive interventions in high-risk individuals, and investigate whether UGT2B15 polymorphisms influence HCC susceptibility in aflatoxin-endemic regions. These findings lay a crucial foundation for further exploration of UGT functions in HCC.

## Data Availability

The original contributions presented in this study are included in the article/Supplementary Materials. The targeted sequencing data have been deposited in the National Center for Biotechnology Information dbGAP database under accession number SRP097455. The microarray data have been deposited in the NCBI GEO database under accession number GSE97387. Further inquiries can be directed to the corresponding authors.

## Supplementary Materials

The following supporting information can be downloaded at https://www.mdpi.com/article/10.3390/cells15090824/s1, Figure S1: The pairwise correlation of UGTs between protein reduction ratio and mRNA expression fold change (UGT1A4, UGT2B4, UGT2B10, UGT2B10); Figure S2: AR upregulates its downstream genes UGT2B15 and UGT2B17 in HCC; Figure S3: The Effects of CCl_4_ or EtOH on the Transcriptome of Liver Cancer Cells; Table S1: Characteristics of 38 HCC patients; Table S2: Oligonucleotide PCR primer for qRT-PCR analysis; Table S3: High confidence somatic non-synonymous mutations identified by targeted sequencing; Table S4: 532 common DGEs between two shUGT2B15 vs shControl comparisons by Agilent SurePrit G3 Human Expression Microarray; Table S5: KEGG pathway enrichment of DEGs.

## Abbreviations

HCC	hepatocellular carcinoma;
UGT	uridine diphospho-glucuronosyltransferase;
DHT	5-dihydrotestosterone;
AR	androgen receptors;
TCGA	The Cancer Genome Atlas;
GWAS	genome-wide association studies;
FLU	4-hydroxy-flutamide;
LC-MS/MS	liquid chromatography–mass spectrometry/mass spectrometry;
HLM	human liver microsomes;
PDX	patient-derived tumor xenograft;
AI	allelic imbalance;
DEGs	differentially expressed genes;
GO	gene ontology.
