# Supplementary material for: UGT2B15 Acts as a Critical Detoxification Barrier Against Chemi-Cal-Induced Hepatotoxicity and Carcinogenesis via the Androgen Receptor Axis
Source: Cells. 2026 Apr 30;15(9):824. doi: 10.3390/cells15090824 (PMC13162611; doi:10.3390/cells15090824)
Supplement: Supplementary file 1 [file cells-15-00824-s001.zip › Supplement Meterial V2_FIN..pdf]

Supplementary Materials for

UGT2B15 acts as a critical detoxification barrier against chemical-induced hepatotoxicity and carcinogenesis via the androgen receptor axis

1. DNA targeted sequencing

1.1 *DNA extraction and sequencing libraries preparation*

The Ion AmpliSeq Library Kit 2.0 (Life Technologies; Part #4475345 Rev. A, Grand Island, NY, USA) was used to construct an Ion Torrent adapter-ligated library as per manufacturer's instructions. Enzymatic shearing of pooled amplicons, adaptor ligation, nick repair and amplification was performed using the Ion XpressTM Plus Fragment Library Kit (Life Technologies, Grand Island, NY, USA). The sheared DNA was further ligated with common adaptors for single samples and Ion X pressTM barcode adapters (Ion Torrent, Life Technologies, Grand Island, NY, USA) for multiplex samples. These libraries were further amplified for 4-6 cycles of PCR. The concentration was further confirmed by a TaqMan Quantitation Kit (Ion Torrent, Life Technologies, Grand Island, NY, USA). The clonal amplification (template preparation) was performed on Ion OneTouch Emulsion PCR using the Ion OneTouchTM 200 Template Kit/Template Kit v2 DL (Ion Torrent, Life Technologies, Grand Island, NY, USA). This was followed by a streptavidin-coupled Dynabeads-based enrichment on an Ion OneTouch Enrichment System (Ion Torrent, Life Technologies, Grand Island, NY, USA) prior to sequencing.

1.2 *Targeted sequencing and bioinformatics analysis*

The Ion Torrent platform-specific pipeline software Torrent Suite 5.0 was used to initially process data from the PGM runs and generate clean reads via trimming adapter sequences, filtering, and removing poor signal profile reads. The clean reads were then mapped to hg19 reference by Torrent Suite Software v3.4 with a plug-in variant caller v3.4. The calling variants were subsequent filtered with multiple thresholds: an average total coverage depth > 100, each variant coverage > 20, variant frequency > 5%, and *P*-value (Fisher exact test) < 0.01. Somatic mutations were obtained by utilizing tumor normal paired analysis by default parameter of Torrent Suite Software. Subsequently, somatic variants were further filtered to get high-confidence somatic variants via removing synonymous, non-coding variants and common SNPs present in UCSC by Ion Reporter (Thermo Fisher, MA, USA).

2. Selection of Signature Peptides for UGTs Quantification

All the signature peptides were synthesized by APeptide Co., Ltd. (Shanghai, China) and their purity (>95%) was determined using HPLC-UV (with a detection wavelength of 220 nm) analysis and ESI-TOF MS analysis. The net peptide content in each stock solution was determined by using quantitative amino acid analysis reported previously(22). All samples were analyzed by using a triple quadruple mass spectrometer (5,500 QTrap, AB Sciex, Foster City, CA) coupled to a Waters Acquity UPLC system (Waters, Milford, MA, USA). The liquid chromatography separation was carried out on an Acquity UPLC HSS T3 column (1.8 μm, 2.1×100 mm). Sample volume of 10 μl was injected to the column and flow rate was 0.5 ml/min. Sample rack and column temperatures were maintained at 10℃ and 40℃, respectively. Mobile phase A was high purity HPLC-grade water with 0.1% (v/v) acetic acid, whereas mobile phase B was 100% acetonitrile with 0.1% (v/v) acetic acid. A linear gradient was used to achieve the chromatographic separation, starting from 5% B and progressing to 30% B over a period of 14 min and regressing to 5% B in 2 min for column equilibration. Quantification was performed in positive ion scheduled MRM mode on an API 5,500 Qtrap triple quadruple mass spectrometer equipped with a Turbo IonSpray TM. The ion counts in the chromatograms were determined using data acquisition software

supplied by AB Sciex (Analyst software 1.5.3). Measured peptide concentrations were converted to protein levels (pmol/mg microsomal protein) based on the standards curve used in the analysis.

3. RNA Isolation from Tissue samples and Quantitative Real-time PCR

Quantitative real-time RT-PCR assay was performed as our previous report(22). Briefly, total RNA was extracted by using TRIzol reagent (Invitrogen, CLD, USA). The cDNA was generated by reverse transcription kit (TaKaRa, Dalian, China) according to the manufacturer's instructions. Target genes were amplified using ABI 7500 fast system (Applied Biosystems, CA, USA). Primer sequences for specific genes were listed in Supplementary Table 2.

4. MTT assay

Huh7 cells were harvested at 72 h after transfection with shControl or shUGT2B15 (8.0×10^3/well in 96-well plate). After adding MTT reagent (0.5 mg/ml) for 4 h incubation, the formazan was dissolved with DMSO. The OD value was read by Victor X3 (PerkinElmer, MA, USA) at 490 nm.

5. Western blot

The protein was centrifuged from cell extraction lysed by RIPA buffer (1% PMSF). Protein concentration was determined using Bradford reagent (Sigma-Aldrich, San Luis, MO, USA). According to the standard WB procedure, the blots were incubated with primary antibodies against AR (1:2000), UGT2B15(1:2000), GAPDH (1:2000) for overnight at 4℃. After incubation with the corresponding secondary antibodies for 1h, the densities of bands were examined by ECL chemiluminescence detection regents (PE, CA, USA). The protein bands were quantified using Quantity-One software (NIH) 4.3.0, and the results were from at least three independently repeated experiments.

6. Transwell assays

Huh7 cells were harvested at 72 h after transfection with shRNA (2×10^4 cells/well) and cultured in the upper chamber with 5% FBS medium, respectively, while high-serum medium (20% FBS) was added to the lower chamber. After 48 h of incubation, cells were fixed with 4 % paraformaldehyde, and stained with 0.1 % crystal violet for 15 min. Then stained cells were dissolved in the DMSO, and the OD value were detected by Victor X3 (PerkinElmer, MA, USA) at 600 nm.

7. Immunofluorescence

According to the previous reported protocol (22), the immunofluorescence assays were performed in MHCC-97H cells with anti-AR (Abcam, 1:200). Cells were incubated with the corresponding TR conjugated secondary antibodies (Santa Cruz, 1:200), following by stained the nucleus with 0.1% DAPI for 30min, then the images were detected by confocal microscopy (Leica, Jena, Germany).

8. Flow cytometry analysis

Apoptosis was detected by Flow cytometry using the Annexin-V/PI Apoptosis Detection Kit followed by manufacture's protocol (Sigma, STL, USA). For each experiment, 10,000 cells were analyzed by flow cytometry (BD Biosciences, San Diego, CA, USA). For cell cycle analysis, cells were harvested, fixed with 70% cold ethanol, incubated with RNase, and stained with propidium iodide (PI). Then stained cells was detected by flow cytometry and analyzed by FlowJo v7.6 software.

9. Gene expression microarray

9.1 ***RNA extraction and purification***

Total RNA was extracted using TRIZOL Reagent (Cat#15596-018, Life technologies, Carlsbad, CA, US) following the manufacturer's instructions and checked for a RIN number to inspect RNA integrity by an Agilent Bioanalyzer 2100 (Agilent technologies, Santa Clara, CA, US). Qualified total RNA was further purified by RNeasy mini kit (Cat#74106, QIAGEN, GmBH, Germany) and RNase-Free DNase Set (Cat#79254, QIAGEN, GmBH, Germany).

9.2 ***RNA amplification and labeling***

Total RNA was amplified and labeled by Low Input Quick Amp Labeling Kit, One-Color (Cat.# 5190-2305, Agilent technologies, Santa Clara, CA, US), following the manufacturer's instructions. Labeled cRNA were purified by RNeasy mini kit (Cat.# 74106, QIAGEN, GmBH, Germany).

9.3 ***Gene expression microarray hybridization***

Each slide was hybridized with 600 ng Cy3-labeled cRNA using Gene Expression Hybridization Kit (Cat.# 5188-5242, Agilent technologies, Santa Clara, CA, US) in Hybridization Oven (Cat.# G2545A, Agilent technologies, Santa Clara, CA, US), according to the manufacturer's instructions. After 17 hours hybridization, slides were washed in staining dishes (Cat.# 121, Thermo Shandon, Waltham, MA, US) with Gene Expression Wash Buffer Kit(Cat.# 5188-5327, Agilent technologies, Santa Clara, CA, US), followed the manufacturer's instructions.

9.4 ***Data acquisition***

Slides were scanned by Agilent Microarray Scanner (Cat#G2565CA, Agilent technologies, Santa Clara, CA, US) with default settings, Dye channel: Green, Scan resolution=3μm, PMT 100%, 20bit. Data were extracted with Feature Extraction software 10.7 (Agilent technologies, Santa Clara, CA, US). Raw data were normalized by Quantile algorithm, limma packages in R.

Supplementary Figure Legends

Figure S1. The pairwise correlation of UGTs between protein reduction ratio and mRNA expression fold change (UGT1A4, UGT2B4, UGT2B10, UGT2B10).

Figure S2. AR upregulates its downstream genes UGT2B15 and UGT2B17 in HCC. (A) Protein level of UGT2B15 in wild-type and mutant tumor cases as detected by LC/MS-MS. **(B)** The pairwise correlation of UGT2B15 between protein expression level reduction ratio and reduction ratio of metabolic activity in tumor versus normal tissues. **(C)** mRNA levels of UGT2B15, UGT2B17 were assessed by qRT-PCR in MHCC-97H cells expressing shControl, shUGT2B15 or UGT2B15 in the presence of DHT (10 nM), or vehicle (0.1%DMSO) for 24h. GAPDH was used as the internal control. Data represent mean ± SD (n=3). *, $P<0.05$; **, $P<0.01$; ***, $P<0.001$. **(D)** mRNA expression levels of AR, UGT2B15, and UGT2B17 genes in a panel of liver cell lines. GAPDH was used for normalization. Representative data from three independent experiments are shown as the mean ± S.D. *, $P<0.05$; **, $P<0.01$; ***, $P<0.001$.

Figure S3. **The Effects of CCl_4 or EtOH on the Transcriptome of Liver Cancer Cells. (A)** Cell viability of MHCC-97H expressing shUGT2B15, UGT2B15 or shControl after exposure to CCl_4 (left) or EtOH (right) as measured by the MTT assay. The data represent the mean ± SD, n=3. Significant differences are indicated by *$P < 0.05$, **$P<0.01$, and ***$P<0.001$. **(B, C)** Protein level validation of selected differentially expressed chemical carcinogenesis metabolism related genes in MHCC-97H cells treated with CCl_4 **(B)** or EtOH **(C)**. GAPDH was used for normalization. Representative data from three independent experiments are shown as the mean ± S.D. *, $P<0.05$; **, $P<0.01$; ***, $P<0.001$.

Supplementary Table S1: Characteristics of 38 HCC patients

**Age at Surgery (Median, Range)**	**50 (23-73)**
**<60 years**	30 (78.94%)
**60 years**	8 (21.06%)
**Gender**	
**Male**	36 (94.73%)
**Female**	2 (5.27%)
**Etiology of the liver disease**	
**HBV***	34 (89.47%)
**HCV**	2 (5.3%)
**AI**	1 (2.63%)
**NVNA**	1 (2.63%)
**Tumor size**	
**≤ 5cm**	10 (26.32%)
**>5cm**	28 (73.68%)
**Serum AFP level**	
**<200**	19 (50%)
**≥200**	19 (50%)
**Edmondson-Steiner grade**	
**1,2**	25 (65.79%)
**3,4**	13(34.21%)
**Tumor numbers**	
**Single**	29 (76.32%)
**Multiple 13 (5.63%)**	9 (23.68%)

*HBV: hepatitis B virus.
HCV: Hepatitis C Virus
AI: alcohol intake
NVNA:non-viral/non-alcoholic causes

Supplementary Table S2: Oligonucleotide PCR primer for qRT-PCR analysis

Gene	species	Types	Sequences	Transcript_ID	No. transcript	Corresponding protein	LC/MS protein
UGT1A1	Human	Forward	TTCTGTGCGACGTGGTTTAT	NM_000463.2	1	NP_000454.1	NP_000454.1
		Reverse	GACAGATGCAGAGCTCAATAGG				
UGT1A3	Human	Forward	GGGACTTTGTGATGGACTACC	NM_019093.2	1	NP_061966.1	NP_061966.1
		Reverse	CTGAGATAGTGGCTTCCTGTTG				
UGT1A4	Human	Forward	CCTGTCCTACATTTGCCATACT	NM_007120.2	1	NP_009051.1	NP_009051.1
		Reverse	CGGATGCATAGCTGACAAGA				
UGT1A6	Human	Forward	GGGACACCCTGAACTTCTTT	NM_001072.3	2	NP_001063.2	NP_001063.2
		Reverse	ACAGATGGTAGGCCCAAATAC				
UGT1A9	Human	Forward	GTTGTTGCGAACGGACTTTG	NM_021027.2	1	NP_066307.1	NP_066307.1
		Reverse	CTTTCCCTGATGGCAGTTGATA				
UGT2B4	Human	Forward	ACTTCAGCTCTTCTGCTGATAC	NM_021139.2	3	NP_066962.2	NP_066962.2
		Reverse	GACCTCTCTGGACAAGTTCATC	NM_001297615.1		NP_001284544.1	NP_001284544.1
UGT2B7	Human	Forward	TCTGTGGAGATTTGATGGGAATAA	NM_001074.3	3	NP_001065.2	NP_001065.2
		Reverse	CTGGTCTTTGGATGACCTAGAAG	NM_001349568.1		NP_001336497.1	
UGT2B10	Human	Forward	GCTGCTGGCTGAGCTATTTA	NM_001075.5	3	NP_001066.1	NP_001066.1
		Reverse	AATCCTCCACTGTGCCTTTC				NP_001138239.1
UGT2B15	Human	Forward	TCTGGCAGATGCCCTTAATC	NM_001076.3	1	NP_001067.2	NP_001067.2
		Reverse	TAGCCAACAGAGAATCGAAGAC				
UGT2B17	Human	Forward	TGCTGGCTGAGCTACTTAAC	NM_001077.3	1	NP_001068.1	NP_001068.1
		Reverse	CATAGGAAGGAGGGAACAGAAAT				
GAPDH	Human	Forward	GGCCTCCAAGGAGTAAGACC	NM_002046.5	4	NP_002037.2	NA
		Reverse	AGGGGAGATTCAGTGTGGTG				
AR	Human	Forward	CCAGATCAGGGTTGAAGAGAAA	NM_000044.4	5	NP_000035.2	NA
		Reverse	ATATGCTGGGTGACAAGAAGAG				

ADH1A	Human	Forward	TAAAGTCATCCCACTCGCTATTC	NM_000667.3	1	NP_000658.1	NA
		Reverse	CCTGAGGATTGCTTACATCGT				
ADH1C	Human	Forward	AGCTGTGCTATGGGAGTTAAAG	NM_000669.4	1	NP_000660.1	NA
		Reverse	CCTGCAGCCACCATCTTAAT				
CYP1B1	Human	Forward	CTGTCTTGGGCTACCACATT	NM_000104.3	1	NP_000095.2	NA
		Reverse	GGATCAAAGTTCTCCGGGTTAG				
MUC1	Human	Forward	CGTGGAGACACAGTTCAATCA	NM_001018016.2	20	NP_001018016.1	NA
		Reverse	GACTGGGCAGAGAAAGGAAAT				
PTGS2	Human	Forward	CTATGTGCTAGCCCACAAAGA	NM_000963.3	1	NP_000954.1	NA
		Reverse	GCATCCACAGATCCCTCAAA				

Supplementary Table S3: High confidence somatic non-synonymous mutations identified by targeted sequencing

Samples	Locus	Ref	Genotype	Genes	Coding	Amino Acid Change	Variant Effect	Allele Ratio	Validation
HCC01	chr2:234627685	A	A/C	UGT1A10,UGT1A4,UGT1A5,UGT1A6, UGT1A7,UGT1A8,UGT1A9	c.219A>C	p.Lys73Asn	missense	A=0.8619, C=0.1381	-
HCC01	chr2:234627701	G	G/A	UGT1A10,UGT1A4,UGT1A5,UGT1A6, UGT1A7,UGT1A8,UGT1A9	c.235G>A	p.Ala79Thr	missense	G=0.837, A=0.163	-
HCC01	chr2:234627709	TG	TG/CA	UGT1A10,UGT1A4,UGT1A5,UGT1A6, UGT1A7,UGT1A8,UGT1A9	c.243_244delTGinsCA	p.Val82Ile	synonymous, missense	TG=0.8424, CA=0.1576	-
HCC01	chr2:234627713	C	C/T	UGT1A10,UGT1A4,UGT1A5,UGT1A6, UGT1A7,UGT1A8,UGT1A9	c.247C>T	p.Pro83Ser	missense	C=0.8378, T=0.1622	-
HCC01	chr2:234628053	AG	AG/GA	UGT1A10,UGT1A4,UGT1A5,UGT1A6, UGT1A7,UGT1A8,UGT1A9	c.587_588delAGinsGA	p.Lys196Arg	missense	AG=0.8788, GA=0.1212	-
HCC01	chr2:234628316	G	G/A	UGT1A10,UGT1A4,UGT1A5,UGT1A6, UGT1A7,UGT1A8,UGT1A9	c.850G>A	p.Gly284Arg	missense	G=0.7857, A=0.2143	-
HCC01	chr2:234638235	A	A/T	UGT1A10,UGT1A3,UGT1A4,UGT1A5, UGT1A6,UGT1A7,UGT1A8,UGT1A9	c.463A>T	p.Asn155Tyr	missense	A=0.9286, T=0.0714	-
HCC01	chr2:234638245	C	C/G	UGT1A10,UGT1A3,UGT1A4,UGT1A5, UGT1A6,UGT1A7,UGT1A8,UGT1A9	c.473C>G	p.Ala158Gly	missense	C=0.8571, G=0.1429	-
HCC01	chr2:234638448	G	G/A	UGT1A10,UGT1A3,UGT1A4,UGT1A5, UGT1A6,UGT1A7,UGT1A8,UGT1A9	c.676G>A	p.Ala226Thr	missense	G=0.8571, A=0.1429	-
HCC01	chr4:69512880	A	A/C	UGT2B15	c.1535T>G	p.Phe512Cys	missense	A=0.843, C=0.157	-
HCC01	chr4:69535975	T	T/G	UGT2B15	c.362A>C	p.Tyr121Ser	missense	T=0.8585, G=0.1415	-

HCC01	chr4:69536246	T	T/C	UGT2B15	c.91A>G	p.Thr31Ala	missense	T=0.8963, C=0.1037	-
HCC01	chr4:69681864	A	A/G	UGT2B10	c.127A>G	p.Lys43Glu	missense	A=0.8667, G=0.1333	-
HCC01	chr4:69681866	A	A/T	UGT2B10	c.129A>T	p.Lys43Asn	missense	A=0.9, T=0.1	-
HCC01	chr4:69682405	T	T/A	UGT2B10	c.668T>A	p.Phe223Tyr	missense	T=0.8857, A=0.1143	-
HCC01	chr4:69683776	A	A/G	UGT2B10	c.748A>G	p.Arg250Gly	missense	A=0.9417, G=0.0583	-
HCC01	chr4:69696354	A	A/T	UGT2B10	c.1344A>T	p.Gln448His	missense	A=0.902, T=0.098	-
HCC01	chr4:69696413	G	G/C	UGT2B10	c.1403G>C	p.Arg468Pro	missense	G=0.95, C=0.05	-
HCC01	chr4:69696590	GG	GG/AT	UGT2B10	c.1580_1581delGGinsAT	p.Arg527Asn	missense	GG=0.8785, AT=0.1215	-
HCC01	chr4:69962326	G	G/C	UGT2B7	c.88G>C	p.Ala30Pro	missense	G=0.8881, C=0.1119	-
HCC01	chr4:69962377	A	A/G	UGT2B7	c.139A>G	p.Ile47Val	missense	A=0.8542, G=0.1458	-
HCC01	chr4:69962836	G	G/A	UGT2B7	c.598G>A	p.Glu200Lys	missense	G=0.8958, A=0.1042	-
HCC01	chr4:70361450	C	C/T	UGT2B4	c.130G>A	p.Asp44Asn	missense	C=0.8848, T=0.1152	-
HCC02	chr2:234638290	T	T/G	UGT1A10,UGT1A3,UGT1A4,UGT1A5, UGT1A6,UGT1A7,UGT1A8,UGT1A9	c.518T>G	p.Leu173Trp	missense	T=0.8923, G=0.1077	-

HCC02	chr4:69433537	G	G/T	UGT2B17	c.666C>A	p.Asp222Glu	missense	G=0.9556, T=0.0444	-
HCC02	chr4:69433638	G	G/T	UGT2B17	c.565C>A	p.Leu189Met	missense	G=0.9675, T=0.0325	-
HCC02	chr4:69681891	G	G/A	UGT2B10	c.154G>A	p.Val52Met	missense	G=0.9516, A=0.0484	-
HCC02	chr4:69681895	C	C/T	UGT2B10	c.158C>T	p.Thr53Ile	missense	C=0.9669, T=0.0331	-
HCC03	chr4:69416506	T	T/G	UGT2B17	c.1202A>C	p.His401Pro	missense	T=0.8333, G=0.1667	-
HCC03	chr4:69681864	A	A/G	UGT2B10	c.127A>G	p.Lys43Glu	missense	A=0.9651, G=0.0349	-
HCC03	chr4:69696568	A	A/G	UGT2B10	c.1558A>G	p.Arg520Gly	missense	A=0.5789, G=0.4211	-
HCC03	chr4:69885420	T	T/C	UGT2B10	c.649A>G	p.Asp216Gly	missense	T=0.4888, C=0.5112	-
HCC04	chr2:234637884	G	G/A	UGT1A10,UGT1A3,UGT1A4,UGT1A5 ,UGT1A6,UGT1A7,UGT1A8,UGT1A9	c.112G>A	p.Gly38Ser	missense	G=0.6604, A=0.3396	-
HCC04	chr4:69416506	T	T/G	UGT2B17	c.1202A>C	p.His401Pro	missense	T=0.8824, G=0.1176	-
HCC04	chr4:69535766	G	G/A	UGT2B15	c.571C>T	p.Pro191Ser	missense	G=0.929, A=0.071	-
HCC04	chr4:69535772	G	G/T	UGT2B15	c.565C>A	p.Leu189Met	missense	G=0.9583, T=0.0417	-
HCC05	chr4:69535775	A	A/G	UGT2B15	c.562T>C	p.Phe188Leu	missense	A=0.8947, G=0.1053	-

HCC05	chr4:69696568	A	A/G	UGT2B10	c.1558A>G	p.Arg520Gly	missense	A=0.5671, G=0.4329	-
HCC05	chr4:69885420	T	T/C	UGT2B10	c.649A>G	p.Asp216Gly	missense	T=0.5443, C=0.4557	-
HCC06	chr4:69416506	T	T/G	UGT2B17	c.1202A>C	p.His401Pro	missense	T=0.8, G=0.2	-
HCC06	chr4:69416528	T	T/C	UGT2B17	c.1180A>G	p.Ile394Val	missense	T=0.9189, C=0.0811	-
HCC07	chr2:234669715	T	T/C	UGT1A1,UGT1A10,UGT1A3,UGT1A4, UGT1A5,UGT1A6,UGT1A7,UGT1A8,UGT1A9	c.782T>C	p.Val261Ala	missense	T=0.9571, C=0.0429	-
HCC07	chr4:69416506	T	T/G	UGT2B17	c.1202A>C	p.His401Pro	missense	T=0.8421, G=0.1579	-
HCC07	chr4:69416528	T	T/C	UGT2B17	c.1180A>G	p.Ile394Val	missense	T=0.8846, C=0.1154	-
HCC07	chr4:69681891	G	G/A	UGT2B10	c.154G>A	p.Val52Met	missense	G=0.9286, A=0.0714	-
HCC07	chr4:69874633	T	T/G	UGT2B10	c.1218A>C	p.Asn406His	missense	T=0.9685, G=0.0315	-
HCC10	chr4:69519866	T	T/G	UGT2B15	c.1202A>C	p.His401Pro	missense	T=0.9452, G=0.0548	-
HCC13	chr4:69681891	G	G/A	UGT2B10	c.154G>A	p.Val52Met	missense	G=0.9107, A=0.0893	YES
HCC13	chr4:69682405	T	T/A	UGT2B10	c.668T>A	p.Phe223Tyr	missense	T=0.9677, A=0.0323	NO
HCC13	chr4:69682416	A	A/G	UGT2B10	c.679A>G	p.Asn227Asp	missense	A=0.9681, G=0.0319	YES

HCC15	chr4:69433518	C	C/T	UGT2B17	c.685G>A	p.Asp229Asn	missense	C=0.8182, T=0.1818	YES
HCC15	chr4:69519971	T	T/C	UGT2B15	c.1097A>G	p.His366Arg	missense	T=0.7035, C=0.2965	NO
HCC15	chr4:69962707	A	A/G	UGT2B7	c.469A>G	p.Ser157Gly	missense	A=0.896, G=0.104	YES
HCC21	chr4:69535772	G	G/T	UGT2B15	c.565C>A	p.Leu189Met	missense	G=0.9658, T=0.0342	YES
HCC21	chr4:69874780	T	T/C	UGT2B10	c.1071A>G	p.Arg357Gly	missense	T=0.96, C=0.04	NO
HCC21	chr4:69879755	T	T/G	UGT2B10	c.954A>C	p.Lys318Gln	missense	T=0.8911, G=0.1089	YES
HCC25	chr4:69535766	GG	GG/AG	UGT2B15	c.571C>T	p.Pro191Ser	missense	GG=0.8947, AG=0.1053, CC=0.0	NO
HCC25	chr4:69536044	A	A/G	UGT2B15	c.293T>C	p.Ile98Thr	missense	A=0.8621, G=0.1379	YES
HCC27	chr4:69519866	T	T/G	UGT2B15	c.1202A>C	p.His401Pro	missense	T=0.9605, G=0.0395	YES
HCC27	chr4:69874623	G	G/A	UGT2B10	c.1228C>T	p.Ser409Leu	missense	G=0.9455, A=0.0545	YES
HCC28	chr4:69519866	T	T/G	UGT2B15	c.1202A>C	p.His401Pro	missense	T=0.958, G=0.042	YES
HCC29	chr4:69416506	T	T/G	UGT2B17	c.1202A>C	p.His401Pro	missense	T=0.8636, G=0.1364	YES

HCC30	chr4:69535717	A	A/G	UGT2B15	c.620T>C	p.Ile207Thr	missense	A=0.9651, G=0.0349	NO
HCC30	chr4:69535769	A	A/G	UGT2B15	c.568T>C	p.Phe190Leu	missense	A=0.9639, G=0.0361	NO
HCC33	chr4:69874626	A	A/G	UGT2B10	c.1225T>C	p.Met408Thr	missense	A=0.9388, G=0.0612, T=0.0	YES
HCC33	chr4:70361228	T	T/C	UGT2B4	c.352A>G	p.Thr118Ala	missense	T=0.9545, C=0.0455	NO
HCC34	chr2:234628203	T	T/C	UGT1A10,UGT1A4,UGT1A5,UGT1A6, UGT1A7,UGT1A8,UGT1A9	c.737T>C	p.Val246Ala	missense	T=0.9008, C=0.0992	YES
HCC34	chr4:69403492	G	G/A	UGT2B17	c.1444C>T	p.His482Tyr	missense	G=0.9444, A=0.0556	YES
HCC34	chr4:69433518	C	C/T	UGT2B17	c.685G>A	p.Asp229Asn	missense	C=0.6429, T=0.3571	YES
HCC34	chr4:69535766	G	G/A	UGT2B15	c.571C>T	p.Pro191Ser	missense	G=0.9298, A=0.0702	YES
HCC34	chr4:69681936	G	G/A	UGT2B10	c.199G>A	p.Asp67Asn	missense	G=0.9013, A=0.0987	YES
HCC34	chr4:69692161	G	G/A	UGT2B10	c.1033G>A	p.Ala345Thr	missense	G=0.5779, A=0.4221	YES
HCC34	chr4:69885505	T	T/C	UGT2B10	c.564A>G	p.Lys188Glu	missense	T=0.9659, C=0.0341	YES
HCC34	chr4:69962723	A	A/C	UGT2B7	c.485A>C	p.Glu162Ala	missense	A=0.97, C=0.03	YES

HCC34	chr4:69962878	A	A/G	UGT2B7	c.640A>G	p.Met214Val	missense	A=0.8752, G=0.1248	YES
HCC34	chr4:69962881	A	A/C	UGT2B7	c.643A>C	p.Ile215Leu	missense	A=0.8679, C=0.1321	YES
HCC34	chr4:70361012	G	G/A	UGT2B4	c.568C>T	p.Pro190Ser	missense	G=0.7722, A=0.2278	YES
HCC35	chr4:69682405	T	T/A	UGT2B10	c.668T>A	p.Phe223Tyr	missense	T=0.9524, A=0.0476	NO
HCC35	chr4:69978320	T	T/A	UGT2B7	c.1456T>A	p.Phe486Ile	missense	T=0.894, A=0.106	YES
HCC36	chr4:69433518	C	C/T	UGT2B17	c.685G>A	p.Asp229Asn	missense	C=0.8333, T=0.1667	YES
HCC36	chr4:69535772	G	G/T	UGT2B15	c.565C>A	p.Leu189Met	missense	G=0.9545, T=0.0455	YES
HCC37	chr4:69513078	A	A/G	UGT2B15	c.1337T>C	p.Leu446Ser	missense	A=0.963, G=0.037	YES
HCC39	chr2:234581389	T	T/G	UGT1A10,UGT1A8,UGT1A9	c.809T>G	p.Ile270Ser	missense	T=0.9681, G=0.0319	YES
HCC39	chr4:69513078	A	A/G	UGT2B15	c.1337T>C	p.Leu446Ser	missense	A=0.9444, G=0.0556	YES
HCC39	chr4:69874633	T	T/G	UGT2B10	c.1218A>C	p.Asn406His	missense	T=0.9552, G=0.0448	YES
HCC40	chr2:234601669	T	T/G	UGT1A10,UGT1A6,UGT1A7,UGT1A8, UGT1A9	c.19T>G	p.Ser7Ala	missense	T=0.8167, G=0.1833	YES
HCC40	chr2:234602191	A	A/G	UGT1A10,UGT1A6,UGT1A7,UGT1A8, UGT1A9	c.541A>G	p.Thr181Ala	missense	A=0.8395, G=0.1605	YES

HCC40	chr2:234602202	A	A/C	UGT1A10,UGT1A6,UGT1A7,UGT1A8, UGT1A9	c.552A>C	p.Arg184Ser	missense	A=0.8393, C=0.1607	YES
HCC40	chr2:234627608	T	T/G	UGT1A10,UGT1A4,UGT1A5,UGT1A6, UGT1A7,UGT1A8,UGT1A9	c.142T>G	p.Leu48Val	missense	T=0.8384, G=0.1616	YES
HCC40	chr2:234637789	A	A/G	UGT1A10,UGT1A3,UGT1A4,UGT1A5, UGT1A6,UGT1A7,UGT1A8,UGT1A9	c.17A>G	p.Gln6Arg	missense	A=0.8838, G=0.1162	NO
HCC40	chr2:234637803	T	T/C	UGT1A10,UGT1A3,UGT1A4,UGT1A5, UGT1A6,UGT1A7,UGT1A8,UGT1A9	c.31T>C	p.Trp11Arg	missense	T=0.8965, C=0.1035	YES
HCC40	chr2:234669144	G	G/A	UGT1A1,UGT1A10,UGT1A3,UGT1A4, UGT1A5,UGT1A6,UGT1A7,UGT1A8,UGT1A9	c.211G>A	p.Gly71Arg	missense	G=0.816, A=0.184	YES
HCC40	chr4:69433632	G	G/A	UGT2B17	c.571C>T	p.Pro191Ser	missense	G=0.7, A=0.3	YES
HCC40	chr4:69535764	A	A/AT	UGT2B15	c.572_573insA	p.Pro192fs	frameshift Insertion	A=0.9048, AT=0.0952	NO
HCC44	chr4:69535766	G	G/A	UGT2B15	c.571C>T	p.Pro191Ser	missense	G=0.9111, A=0.0889	YES
HCC44	chr4:69681891	G	G/A	UGT2B10	c.154G>A	p.Val52Met	missense	G=0.9318, A=0.0682	YES
HCC45	chr4:69536044	A	A/G	UGT2B15	c.293T>C	p.Ile98Thr	missense	A=0.9633, G=0.0367	YES
HCC45	chr4:69536085	A	A/T	UGT2B15	c.252T>A	p.Asn84Lys	missense	A=0.9478, T=0.0522	NO
HCC45	chr4:69885580	T	T/A	UGT2B10	c.489A>T	p.Thr163Ser	missense	T=0.8333, A=0.1667	YES

Note: -,not validated;

Supplementary Table S4. 532 common DGEs between two shUGT2B15 vs shControl comparisons by Agilent SurePrit G3 Human Expression Microarray.

ProbeName	GeneSymbol	shCtrl_Normalized signal (log2)	shUGT2B15#1_Normalized signal (log2)	Fold Change	shUGT2B15#2_Normalized signal (log2)	Fold Change	Regulation
A_23_P5211	MUC16	2.82	4.36	2.91	4.16	2.54	up
A_24_P237586	ANKRD37	7.38	8.48	2.16	8.57	2.29	up
A_23_P84189	PITPNC1	4.89	6.43	2.91	6.22	2.51	up
A_23_P33881	SSX4B	4.33	5.91	2.97	5.79	2.75	up
A_33_P3385006	SLC39A5	5.87	4.53	0.39	4.59	0.41	down
A_23_P132763	VGLL3	5.10	6.27	2.24	6.38	2.43	up
A_33_P3294986	LIPE	9.19	7.76	0.37	8.06	0.46	down
A_33_P3335590	CCDC64B	7.27	8.44	2.24	8.78	2.84	up
A_33_P3407344	CEP85L	6.94	4.61	0.20	1.92	0.03	down
A_24_P6449	SAMD7	5.86	3.52	0.20	1.89	0.06	down
A_33_P3345314	ZFP64	2.96	4.69	3.32	4.51	2.93	up
A_33_P3712341	CXCL12	5.94	4.82	0.46	4.52	0.37	down
A_24_P192914	AMICA1	8.60	7.58	0.49	7.18	0.37	down
A_23_P24294	SLC17A6	5.06	1.97	0.12	2.61	0.18	down
A_33_P3339246	LHX8	6.20	3.47	0.15	3.82	0.19	down
A_33_P3363515	XRCC3	13.85	15.32	2.77	15.14	2.46	up
A_33_P3214436	TSEN15	4.62	2.55	0.24	2.83	0.29	down
A_23_P150768	SLCO2B1	8.64	10.24	3.04	10.19	2.92	up
A_33_P3297244	GAS2L2	4.91	3.04	0.28	2.91	0.25	down
A_33_P3267989	CLNK	5.13	2.16	0.13	2.26	0.14	down
A_33_P3251751	CMBL	4.77	2.86	0.27	2.45	0.20	down
A_33_P3888485	BCO2	6.34	4.99	0.39	5.31	0.49	down
A_33_P3255290	JAKMIP2	5.32	1.79	0.09	2.20	0.11	down

A_23_P101054	KRT34	2.65	4.50	3.61	4.44	3.44	up
A_33_P3379841	N4BP2L2-IT2	6.75	5.71	0.49	5.38	0.39	down
A_23_P57474	OSBP2	3.59	5.93	5.07	4.92	2.52	up
A_23_P19333	TREM1	4.43	5.65	2.33	6.57	4.40	up
A_32_P41604	F5	6.62	5.13	0.36	5.54	0.47	down
A_24_P236091	ENO2	11.30	12.57	2.40	12.32	2.02	up
A_23_P50250	CKM	3.43	4.88	2.74	4.74	2.48	up
A_23_P77918	HS3ST3B1	4.80	3.29	0.35	2.74	0.24	down
A_23_P140450	SLC27A2	5.90	4.75	0.45	4.80	0.47	down
A_23_P417014	ZNF311	5.45	3.50	0.26	4.38	0.48	down
A_22_P00025517	TLX1NB	6.33	4.33	0.25	4.80	0.35	down
A_22_P00012574	BARX1-AS1	5.23	3.99	0.42	3.49	0.30	down
A_23_P422851	CABLES1	11.51	10.06	0.36	9.98	0.35	down
A_23_P156824	HTR1B	3.76	5.15	2.62	5.09	2.52	up
A_32_P194062	TYR	4.50	1.88	0.16	1.89	0.16	down
A_23_P350001	GUCY1A2	5.05	1.88	0.11	1.93	0.11	down
A_33_P3345717	DCX	4.60	2.95	0.32	3.03	0.34	down
A_24_P945181	RBM15B	3.98	5.07	2.13	5.27	2.44	up
A_24_P339944	PDGFB	7.12	8.23	2.16	8.46	2.54	up
A_23_P105691	GPRC5D	4.86	3.14	0.30	2.26	0.17	down
A_23_P77859	TMEM88	5.60	6.87	2.41	6.71	2.16	up
A_24_P92472	CFI	4.76	5.80	2.05	6.08	2.49	up
A_23_P142738	TMEM178A	4.36	1.79	0.17	1.79	0.17	down
A_21_P0006455	PABPC1L2B-AS1	3.51	5.06	2.93	4.54	2.04	up
A_24_P322771	TFF1	13.51	12.40	0.47	12.39	0.46	down

A_32_P164593	ZMAT4	5.53	4.42	0.46	4.18	0.39	down
A_23_P312752	KCNJ13	6.13	1.98	0.06	2.29	0.07	down
A_24_P323967	ZNF880	5.27	3.45	0.28	2.01	0.10	down
A_23_P19182	REEP2	3.86	5.85	3.97	5.37	2.86	up
A_33_P3215744	PART1	4.86	6.71	3.62	6.96	4.29	up
A_23_P85218	SOX3	3.40	4.86	2.75	5.05	3.13	up
A_21_P0000464	SNORD123	4.86	2.16	0.15	2.07	0.14	down
A_23_P36187	SYT8	2.87	4.85	3.94	4.52	3.13	up
A_23_P49448	FA2H	11.17	9.93	0.42	9.93	0.42	down
A_32_P199551	SPTSSB	7.87	6.78	0.47	5.89	0.25	down
A_23_P104073	S100A3	12.56	13.70	2.21	14.01	2.75	up
A_23_P381505	VWDE	5.99	2.39	0.08	2.14	0.07	down
A_23_P501985	CSF2RA	6.15	1.81	0.05	2.36	0.07	down
A_23_P114134	SSX3	6.09	4.72	0.39	4.89	0.43	down
A_23_P35529	MBL2	5.07	2.44	0.16	4.00	0.48	down
A_23_P154708	HAO1	5.70	2.11	0.08	3.73	0.25	down
A_23_P10542	HTRA3	5.29	6.29	2.00	6.63	2.55	up
A_22_P00012283	PPP2R2D	8.73	7.37	0.39	7.22	0.35	down
A_23_P351148	SH2D1B	3.31	4.68	2.57	5.38	4.18	up
A_33_P3391076	MBNL2	6.59	4.79	0.29	5.34	0.42	down
A_21_P0010517	MST1	7.11	3.39	0.08	3.10	0.06	down
A_23_P97606	GSTM5	6.61	4.80	0.29	4.38	0.21	down
A_23_P122216	LOX	7.37	8.90	2.89	8.74	2.57	up
A_24_P404458	NMRK2	6.59	2.00	0.04	2.06	0.04	down
A_24_P2361	CLCNKA	3.29	4.82	2.88	4.57	2.42	up
A_32_P213459	DMRT2	2.10	4.35	4.78	4.59	5.65	up

A_24_P208567	IL18R1	5.24	4.13	0.46	4.20	0.49	down
A_33_P3354945	CSF1	4.63	3.28	0.39	3.32	0.40	down
A_23_P81650	C5orf15	9.51	10.56	2.07	10.68	2.24	up
A_33_P3266639	TMEM220	4.76	2.22	0.17	2.23	0.17	down
A_33_P3299416	GRASP	6.12	7.36	2.37	7.50	2.60	up
A_33_P3275600	PLA2G4E	5.08	3.77	0.40	3.74	0.40	down
A_23_P212258	KNG1	7.42	5.74	0.31	5.12	0.20	down
A_21_P0009341	WFDC21P	6.62	5.42	0.44	4.00	0.16	down
A_33_P3355659	SKOR1	5.84	1.86	0.06	1.86	0.06	down
A_23_P366098	NUP210L	5.44	1.83	0.08	1.95	0.09	down
A_22_P00000214	LIG3	5.67	3.87	0.29	3.40	0.21	down
A_23_P121851	PCDHB15	4.39	5.71	2.50	5.76	2.58	up
A_33_P3246598	NUGGC	6.39	5.34	0.48	5.35	0.49	down
A_23_P39605	SULT1C4	6.06	3.84	0.22	4.44	0.32	down
A_32_P119033	PLCXD3	5.82	4.52	0.41	4.26	0.34	down
A_23_P27315	EMILIN2	2.99	4.22	2.35	4.82	3.57	up
A_23_P409623	PPFIBP2	8.33	7.31	0.49	6.89	0.37	down
A_32_P74409	C11orf96	6.54	7.62	2.11	7.60	2.07	up
A_23_P76749	GALNT16	2.32	4.53	4.63	4.65	5.01	up
A_33_P3302373	SZT2	3.25	4.59	2.53	4.82	2.97	up
A_23_P133058	MRFAP1L1	8.75	9.86	2.16	9.87	2.17	up
A_33_P3268555	SP140	4.94	7.03	4.26	6.40	2.76	up
A_33_P3542886	SNAR-G1	4.78	3.73	0.49	2.77	0.25	down
A_23_P393025	SPATA4	3.26	4.48	2.34	4.36	2.15	up
A_33_P3376249	S100A2	11.64	12.65	2.02	12.85	2.31	up
A_21_P0000163	RNF223	5.22	6.43	2.32	6.31	2.13	up

A_23_P42868	IGFBP1	12.44	13.57	2.20	14.15	3.27	up
A_33_P3400700	SLC26A5	5.07	1.83	0.11	1.85	0.11	down
A_24_P276576	FCRLA	5.16	1.83	0.10	1.83	0.10	down
A_22_P00025959	PTPN14	4.43	3.40	0.49	3.36	0.48	down
A_33_P3293753	KRTAP10-9	7.59	6.44	0.45	6.56	0.49	down
A_22_P00005046	XKR5	3.10	4.40	2.46	4.39	2.43	up
A_33_P3238280	ESYT3	4.93	2.22	0.15	2.00	0.13	down
A_33_P3342752	CAPN8	5.99	4.72	0.42	4.41	0.34	down
A_32_P44394	AIM2	3.78	5.54	3.38	5.35	2.96	up
A_21_P0013906	TTTY14	5.70	2.07	0.08	2.05	0.08	down
A_23_P5031	LGALS13	6.81	4.06	0.15	1.79	0.03	down
A_23_P351168	DNAJB7	6.17	2.07	0.06	2.07	0.06	down
A_23_P323180	HOXD3	3.71	5.06	2.56	5.14	2.70	up
A_32_P75357	KLHL14	5.30	2.14	0.11	2.57	0.15	down
A_23_P257043	GEM	8.18	9.25	2.10	9.38	2.30	up
A_33_P3419314	CHD5	4.97	6.01	2.06	6.51	2.92	up
A_23_P209944	RETSAT	8.24	7.24	0.50	7.04	0.43	down
A_21_P0000118	LEKR1	3.38	4.50	2.18	4.50	2.18	up
A_32_P481377	KRTAP11-1	4.58	2.51	0.24	3.27	0.40	down
A_24_P144303	XRCC6P5	5.62	1.84	0.07	2.71	0.13	down
A_33_P3220670	APOOP5	5.25	3.32	0.26	3.64	0.33	down
A_23_P210330	LGALSL	11.40	10.33	0.48	10.28	0.46	down
A_23_P256470	NPY	3.30	5.15	3.59	5.30	4.00	up
A_33_P3254431	C1orf86	5.78	4.22	0.34	4.05	0.30	down
A_33_P3239123	PPIH	4.59	2.09	0.18	3.20	0.38	down
A_23_P416666	GJA10	5.81	2.90	0.13	2.01	0.07	down

A_21_P0000021	ADAL	4.11	6.21	4.29	5.35	2.36	up
A_33_P3384133	C17orf99	8.47	6.69	0.29	7.12	0.39	down
A_23_P14716	ADPGK	8.71	9.72	2.01	9.77	2.08	up
A_33_P3332150	APOBEC4	4.43	1.76	0.16	1.76	0.16	down
A_33_P3555368	FLJ26086	5.18	3.06	0.23	3.15	0.24	down
A_22_P00001765	OPA1-AS1	5.77	2.01	0.07	2.37	0.09	down
A_22_P00001448	PRKCA-AS1	5.23	4.06	0.44	3.90	0.40	down
A_23_P300740	NPB	8.83	9.93	2.14	10.61	3.43	up
A_23_P200579	CELA3B	6.55	4.58	0.26	2.07	0.04	down
A_23_P171150	KLHL4	3.89	4.90	2.03	4.92	2.05	up
A_23_P255328	PRDM8	3.61	5.42	3.50	4.81	2.30	up
A_24_P29733	CDK14	6.56	7.83	2.41	7.61	2.07	up
A_24_P932416	TMEM14E	6.66	4.71	0.26	5.01	0.32	down
A_23_P71073	TWIST1	4.55	2.12	0.19	2.07	0.18	down
A_33_P3335730	FLJ16171	5.96	1.83	0.06	3.15	0.14	down
A_33_P3313456	CXorf30	3.03	5.21	4.51	4.17	2.20	up
A_23_P27606	IL27RA	6.64	7.76	2.18	7.66	2.03	up
A_23_P156327	TGFBI	13.42	14.69	2.42	14.85	2.68	up
A_23_P369471	LCE3A	4.60	3.19	0.38	3.15	0.36	down
A_24_P506816	OR2A20P	3.07	5.13	4.17	5.11	4.10	up
A_24_P135193	UEVLD	4.93	3.75	0.44	3.71	0.43	down
A_33_P3412184	ZNF865	6.65	5.49	0.45	5.24	0.38	down
A_23_P371039	NTSR1	6.57	7.66	2.13	7.57	2.00	up
A_21_P0014039	FBRSL1	6.19	7.30	2.16	7.20	2.02	up
A_24_P275073	ADAMTS14	3.81	5.15	2.52	5.46	3.14	up
A_21_P0000428	SNORD114-31	4.86	1.80	0.12	1.81	0.12	down

A_21_P0000793	ZRANB2-AS1	4.70	3.59	0.46	1.82	0.14	down
A_33_P3333554	ACTR3C	4.94	1.98	0.13	3.45	0.36	down
A_23_P27075	GABARAP	12.19	13.26	2.09	13.25	2.08	up
A_33_P3371904	ASTN2	4.84	3.83	0.49	3.51	0.40	down
A_33_P3267320	RFT1	3.13	4.45	2.49	4.48	2.54	up
A_33_P3291579	PAX7	4.38	2.41	0.26	2.61	0.29	down
A_23_P323801	BEST3	5.38	3.61	0.29	2.78	0.17	down
A_23_P215111	ATP6V0A4	5.42	6.76	2.53	7.25	3.56	up
A_33_P3357002	C1QTNF9B	5.66	1.83	0.07	1.83	0.07	down
A_23_P22444	CFP	8.14	6.83	0.40	6.34	0.29	down
A_23_P371145	ADPRHL1	4.52	5.82	2.46	5.87	2.54	up
A_23_P39755	B3GNT7	3.72	4.90	2.27	4.92	2.30	up
A_33_P3221723	MGAT4C	3.63	5.18	2.93	4.90	2.42	up
A_33_P3337272	NRARP	10.17	9.00	0.45	9.14	0.49	down
A_23_P33196	COL5A2	10.38	11.39	2.02	11.43	2.07	up
A_23_P13094	MMP10	5.19	6.45	2.40	6.20	2.02	up
A_23_P26522	AQP8	7.17	6.12	0.48	5.88	0.41	down
A_33_P3379881	FMN1	4.40	1.82	0.17	1.82	0.17	down
A_21_P0007910	DLEU1	5.55	4.30	0.42	3.73	0.28	down
A_33_P3326271	OR13C2	5.82	2.00	0.07	2.05	0.07	down
A_33_P3311353	NUTM1	5.59	2.02	0.08	1.97	0.08	down
A_24_P684183	SLC44A4	5.93	2.78	0.11	3.79	0.23	down
A_23_P319583	RIMS3	7.06	8.26	2.30	8.49	2.68	up
A_23_P212617	TFRC	12.17	13.42	2.37	13.61	2.70	up
A_33_P3346771	AK9	5.21	2.11	0.12	2.74	0.18	down
A_33_P3222977	TEKT5	1.81	4.43	6.16	4.14	5.05	up

A_23_P52986	VWCE	5.39	7.04	3.13	7.31	3.79	up
A_33_P3380625	HSPG2	6.48	4.40	0.24	4.30	0.22	down
A_24_P329229	KCTD7	3.27	4.61	2.52	4.37	2.14	up
A_22_P00016474	CASC8	5.14	1.94	0.11	2.32	0.14	down
A_23_P216340	SLA	5.00	6.59	3.01	6.06	2.08	up
A_33_P3350306	ZNF589	5.46	3.83	0.32	2.30	0.11	down
A_33_P3381191	FOXRED2	7.18	4.35	0.14	4.02	0.11	down
A_33_P3274084	RHBDL3	4.05	5.78	3.31	6.12	4.22	up
A_33_P3343872	SIRPG	5.56	3.55	0.25	3.94	0.33	down
A_23_P365738	ARC	5.63	7.38	3.37	7.27	3.12	up
A_21_P0000882	RNF157-AS1	3.62	6.16	5.82	5.55	3.80	up
A_33_P3259373	PWAR1	4.24	3.13	0.46	2.31	0.26	down
A_33_P3261620	GHSR	3.44	4.45	2.01	4.69	2.37	up
A_23_P102117	WNT10A	5.94	3.60	0.20	4.20	0.30	down
A_22_P00017184	DARS-AS1	5.27	6.41	2.20	6.38	2.16	up
A_33_P3237135	MMP2	5.42	6.49	2.10	6.63	2.32	up
A_22_P00000283	MIR940	4.93	3.43	0.35	3.53	0.38	down
A_23_P331813	ZNF687	8.10	9.24	2.20	9.22	2.17	up
A_22_P00004951	ST3GAL6-AS1	2.81	4.53	3.30	4.91	4.30	up
A_23_P7212	CFI	7.84	5.80	2.05	8.98	2.20	up
A_33_P3398316	MMP27	7.39	2.63	0.04	1.88	0.02	down
A_33_P3355014	TMEM229B	5.05	3.23	0.28	3.86	0.44	down
A_23_P42397	PRSS35	4.87	2.74	0.23	3.61	0.42	down
A_33_P3303649	MB	4.83	3.77	0.48	3.31	0.35	down
A_22_P00009069	FAM183CP	4.99	2.09	0.13	2.05	0.13	down
A_33_P3317508	GPR148	6.14	1.88	0.05	1.89	0.05	down

A_33_P3404316	MIR100HG	5.46	6.89	2.70	6.67	2.32	up
A_32_P505133	C19orf47	4.13	5.20	2.09	5.15	2.03	up
A_23_P323823	HIST1H2BA	6.16	1.79	0.05	2.76	0.09	down
A_23_P301360	ZNF572	5.83	3.15	0.16	1.90	0.07	down
A_23_P48561	EFS	4.94	3.84	0.47	2.61	0.20	down
A_21_P0002253	ST3GAL5-AS1	2.45	4.25	3.49	4.13	3.21	up
A_23_P87036	APOA4	5.98	7.86	3.67	8.97	7.94	up
A_23_P46755	GDF2	6.00	1.85	0.06	4.08	0.26	down
A_24_P208081	PLA2G2F	5.65	4.54	0.46	3.71	0.26	down
A_23_P139527	HPD	6.37	8.40	4.08	10.10	13.21	up
A_23_P153301	CEACAM5	9.77	7.89	0.27	8.04	0.30	down
A_23_P212756	GRK4	4.33	3.18	0.45	3.21	0.46	down
A_33_P3312119	C6orf99	5.58	2.07	0.09	1.96	0.08	down
A_24_P250922	PTGS2	7.30	9.49	4.56	8.63	2.51	up
A_33_P3354344	POM121L10P	4.48	1.94	0.17	1.89	0.17	down
A_23_P162288	MYO1A	7.13	5.77	0.39	6.08	0.48	down
A_33_P3412463	PLEKHS1	6.56	4.61	0.26	2.86	0.08	down
A_33_P3421913	CADM1	4.97	6.06	2.12	6.27	2.46	up
A_33_P3381851	KRTAP10-10	8.16	5.62	0.17	7.07	0.47	down
A_33_P3365582	RASGRP3	4.36	2.65	0.30	2.90	0.36	down
A_19_P00804723	RPS24	3.15	4.25	2.15	4.15	2.01	up
A_33_P3214109	METTL21EP	4.90	2.23	0.16	2.18	0.15	down
A_23_P57784	CLDN1	5.64	4.60	0.48	4.36	0.41	down
A_33_P3350086	OR2T33	4.58	1.78	0.14	1.78	0.14	down
A_24_P346762	FAM214B	5.17	6.26	2.12	6.41	2.35	up
A_33_P3355508	FOXL2	5.58	2.08	0.09	2.00	0.08	down

A_23_P113237	CRLF2	4.71	3.16	0.34	2.07	0.16	down
A_33_P3349637	PCDH1	10.33	9.07	0.42	9.19	0.45	down
A_24_P555170	UBE2Q2L	5.01	6.23	2.33	7.25	4.73	up
A_33_P3319625	NFIA	4.62	3.45	0.44	3.19	0.37	down
A_23_P45955	TEKT2	3.93	5.62	3.22	5.46	2.90	up
A_23_P401606	EDIL3	5.78	2.36	0.09	2.47	0.10	down
A_23_P120504	TMEM74B	5.57	6.79	2.32	7.23	3.15	up
A_24_P355626	ABCG4	4.47	3.27	0.43	2.56	0.27	down
A_24_P339514	CYP2B6	13.07	11.70	0.39	11.38	0.31	down
A_23_P24784	TNNI2	4.26	6.00	3.33	5.69	2.69	up
A_33_P3284345	NRG1	8.38	9.71	2.51	10.27	3.71	up
A_23_P167493	CARTPT	4.49	2.70	0.29	3.30	0.44	down
A_23_P160318	COL16A1	8.36	6.95	0.38	6.60	0.29	down
A_33_P3369158	KIF3C	5.05	6.12	2.10	6.29	2.36	up
A_24_P300302	ZNF300P1	5.42	2.36	0.12	3.42	0.25	down
A_22_P00012501	PAX8-AS1	2.42	4.33	3.74	4.16	3.34	up
A_33_P3230723	KIF1B	3.82	4.90	2.12	4.95	2.19	up
A_33_P3332369	GAD2	4.98	3.79	0.44	2.61	0.19	down
A_24_P328524	KALRN	5.97	7.27	2.47	7.40	2.70	up
A_24_P385732	SLC51A	8.90	7.09	0.29	7.42	0.36	down
A_23_P66593	GFAP	5.30	3.06	0.21	1.99	0.10	down
A_23_P502142	FYN	5.83	1.86	0.06	3.39	0.18	down
A_33_P3280700	GPR157	4.82	2.09	0.15	1.91	0.13	down
A_23_P401700	APBB1IP	8.35	9.64	2.44	9.59	2.35	up
A_33_P3388283	HMGCL	3.04	4.66	3.08	4.17	2.19	up
A_24_P289260	DACT2	8.12	7.06	0.48	6.46	0.32	down

A_23_P3302	MNS1	4.12	5.23	2.15	5.53	2.65	up
A_33_P3251582	LRRC16A	4.98	3.77	0.43	3.71	0.42	down
A_33_P3312384	MIR99AHG	2.73	4.15	2.67	4.96	4.68	up
A_23_P57364	TFF2	11.67	9.52	0.23	9.02	0.16	down
A_24_P240187	LRRN1	5.90	1.93	0.06	1.93	0.06	down
A_23_P58676	NPR3	5.32	4.90	2.78	7.59	4.82	up
A_23_P103349	OCLM	6.03	2.88	0.11	1.93	0.06	down
A_33_P3293888	AFF2	1.80	4.46	6.32	4.66	7.29	up
A_23_P101407	C3	5.08	6.69	3.04	6.11	2.04	up
A_33_P3304668	COL1A1	2.69	4.17	2.79	4.01	2.49	up
A_33_P3265749	PTGER3	3.52	5.35	3.57	4.57	2.06	up
A_23_P111361	PPIL6	5.68	4.05	0.32	4.35	0.40	down
A_33_P3395688	MYZAP	4.64	2.56	0.24	3.26	0.38	down
A_23_P84929	SLC38A5	8.45	10.30	3.60	9.75	2.46	up
A_23_P1173	HABP2	6.53	4.99	0.34	5.45	0.47	down
A_23_P47616	FOLH1	5.09	3.99	0.47	1.71	0.10	down
A_23_P206359	CDH1	8.94	7.24	0.31	7.04	0.27	down
A_23_P70509	ATAT1	3.24	4.85	3.07	4.89	3.14	up
A_24_P262127	RRAD	1.99	5.66	12.76	5.29	9.83	up
A_33_P3313145	ITIH3	5.94	4.61	0.40	4.72	0.43	down
A_23_P64873	DCN	6.94	8.08	2.20	8.46	2.85	up
A_24_P215240	ENKUR	4.71	6.75	4.12	6.11	2.64	up
A_23_P111583	CD36	9.98	8.65	0.40	8.67	0.40	down
A_33_P3394213	GRIN3B	3.86	5.43	2.97	6.17	4.99	up
A_22_P00016229	CATIP-AS2	5.25	2.47	0.15	1.83	0.09	down
A_23_P140760	GPR97	2.44	4.41	3.92	4.13	3.21	up

A_33_P3221418	NTNG1	5.25	1.89	0.10	1.89	0.10	down
A_23_P127107	CLRN3	9.94	8.76	0.44	8.70	0.42	down
A_33_P3342260	GCK	3.41	5.29	3.68	4.99	3.00	up
A_23_P73747	ARMCX2	4.50	5.81	2.47	5.93	2.69	up
A_23_P85922	BMP8A	5.60	1.93	0.08	1.90	0.08	down
A_33_P3295710	OR4K15	4.80	3.04	0.30	1.82	0.13	down
A_33_P3214012	HMGCLL1	5.47	4.26	0.43	4.14	0.40	down
A_23_P125977	C1QC	6.38	1.95	0.05	2.02	0.05	down
A_23_P401106	PDE2A	9.76	11.21	2.75	11.26	2.85	up
A_21_P0006621	CALML3-AS1	5.45	1.83	0.08	1.87	0.08	down
A_21_P0014041	IPO13	4.44	2.04	0.19	2.01	0.19	down
A_33_P3371154	ZNF365	4.60	3.47	0.45	3.00	0.33	down
A_24_P57993	KIAA1644	4.73	1.77	0.13	1.77	0.13	down
A_23_P76218	DCD	5.28	2.11	0.11	2.13	0.11	down
A_33_P3291816	CLDN22	2.42	4.66	4.74	5.11	6.47	up
A_33_P3352073	MBNL1-AS1	4.72	1.96	0.15	1.72	0.13	down
A_24_P937317	IL31	4.55	2.05	0.18	1.81	0.15	down
A_23_P400310	REG4	5.19	3.49	0.31	2.88	0.20	down
A_23_P215484	CCL26	10.20	11.44	2.37	11.86	3.16	up
A_23_P90357	TBXA2R	6.52	7.82	2.46	7.81	2.45	up
A_32_P174693	C1orf234	4.71	2.09	0.16	2.06	0.16	down
A_23_P136371	NDST4	5.09	1.99	0.12	2.06	0.12	down
A_24_P357169	EPPK1	5.80	6.85	2.07	6.85	2.08	up
A_32_P83049	EFR3B	7.42	8.81	2.63	8.94	2.88	up
A_23_P42882	CAMK2B	5.56	4.08	0.36	4.43	0.46	down
A_32_P191441	C10orf90	8.81	10.07	2.39	10.77	3.89	up

A_33_P3382999	PSAPL1	4.47	1.74	0.15	1.74	0.15	down
A_24_P401870	C9orf139	4.94	3.65	0.41	3.29	0.32	down
A_33_P3390217	TTLL9	4.45	5.69	2.35	5.51	2.07	up
A_33_P3369760	GLIPR2	4.17	5.37	2.30	5.53	2.57	up
A_22_P00009941	IKZF3	5.67	2.04	0.08	2.07	0.08	down
A_23_P386888	LPA	5.05	2.80	0.21	2.86	0.22	down
A_33_P3325502	ARHGAP29	3.03	4.69	3.15	4.40	2.58	up
A_23_P114466	TBL1Y	9.05	7.99	0.48	7.95	0.47	down
A_33_P3254460	DLK2	4.82	6.00	2.26	5.94	2.18	up
A_23_P414654	RAB37	9.52	7.69	0.28	8.41	0.47	down
A_21_P0000353	SCARNA23	5.58	3.94	0.32	4.47	0.46	down
A_33_P3310744	TTC34	5.76	3.15	0.16	2.39	0.10	down
A_33_P3216570	MUC5AC	6.22	4.59	0.32	4.54	0.31	down
A_23_P217277	SLITRK2	6.66	2.07	0.04	2.02	0.04	down
A_23_P129005	NYNRIN	3.52	4.74	2.32	4.54	2.02	up
A_33_P6817004	PCBP1-AS1	4.33	2.53	0.29	1.73	0.16	down
A_23_P71037	IL6	4.74	7.17	5.37	7.10	5.13	up
A_23_P10291	CTSE	6.18	4.93	0.42	4.91	0.41	down
A_33_P3290082	HPS4	2.98	4.60	3.06	4.51	2.89	up
A_24_P649282	LUZP2	5.45	4.44	0.50	3.97	0.36	down
A_33_P3316115	ITFG1-AS1	4.35	1.78	0.17	2.11	0.21	down
A_33_P3396404	CLLU1OS	6.06	7.12	2.09	7.20	2.21	up
A_33_P3314176	FAM46C	1.91	4.35	5.43	4.85	7.67	up
A_23_P103588	HMGCS2	7.50	5.41	0.23	5.82	0.31	down
A_33_P3270941	C1orf229	3.05	4.40	2.56	4.36	2.48	up
A_33_P3500631	SLC7A11-AS1	4.80	2.29	0.18	2.21	0.17	down

A_33_P3348937	FLJ42220	4.54	1.84	0.15	1.87	0.16	down
A_33_P3317580	GFRA2	5.05	6.35	2.45	6.67	3.06	up
A_24_P36229	DTNA	2.76	4.40	3.12	5.00	4.72	up
A_23_P160940	ABCA4	8.29	9.59	2.47	9.48	2.28	up
A_33_P3344334	KCNH5	5.08	1.80	0.10	2.07	0.12	down
A_33_P3256282	KRTAP10-2	1.97	5.01	8.22	5.01	8.20	up
A_33_P3213235	CLEC4G	2.87	4.35	2.78	4.46	3.01	up
A_33_P3329088	PRSS8	7.61	6.17	0.37	5.85	0.29	down
A_33_P3279629	UCN2	2.14	5.17	8.13	4.75	6.11	up
A_33_P3384052	RAX	3.05	4.59	2.91	4.20	2.23	up
A_23_P133408	CSF2	4.37	1.87	0.18	1.85	0.17	down
A_24_P53778	ITLN2	6.25	4.01	0.21	4.19	0.24	down
A_23_P133739	HUS1B	3.00	4.26	2.40	4.70	3.26	up
A_33_P3348872	PRDM16	8.08	6.96	0.46	6.67	0.38	down
A_33_P3271594	TRIM54	9.51	10.92	2.67	10.86	2.56	up
A_21_P0011331	CSPG4	3.75	4.82	2.09	4.80	2.07	up
A_23_P111888	CTHRC1	6.22	7.66	2.72	7.65	2.69	up
A_23_P310460	MDGA1	7.26	8.50	2.36	8.51	2.38	up
A_23_P150394	FXYD6	2.90	4.77	3.65	4.52	3.07	up
A_22_P00017060	ADAM8	3.47	4.81	2.53	4.90	2.70	up
A_22_P00020609	VPS13A-AS1	6.32	5.13	0.44	5.19	0.46	down
A_23_P214267	GPR110	9.51	7.63	0.27	8.29	0.43	down
A_23_P127495	BBOX1	9.05	7.64	0.38	7.76	0.41	down
A_33_P3360611	MPP2	3.42	4.95	2.89	4.45	2.04	up
A_23_P897	C1orf116	5.11	3.86	0.42	4.06	0.48	down
A_23_P406458	MRGPRG-AS1	6.53	1.99	0.04	2.03	0.04	down

A_24_P97687	HTR1A	6.06	1.82	0.05	1.83	0.05	down
A_23_P112026	IDO1	5.34	3.09	0.21	2.06	0.10	down
A_24_P313431	CBLN1	4.87	2.09	0.15	2.07	0.14	down
A_23_P72387	AFAP1	5.85	7.12	2.42	7.08	2.36	up
A_24_P16833	FAT3	5.54	1.84	0.08	1.87	0.08	down
A_24_P150068	HTR3A	4.70	2.30	0.19	3.40	0.41	down
A_23_P24234	OPN4	4.79	6.07	2.42	6.21	2.66	up
A_24_P71244	PIK3CD	4.31	5.65	2.54	5.34	2.04	up
A_32_P133840	TMCC2	4.75	5.87	2.18	6.10	2.55	up
A_23_P365614	NOTCH4	1.94	5.40	11.01	4.84	7.47	up
A_23_P115261	AGT	10.54	9.38	0.45	9.45	0.47	down
A_23_P350005	TRIML2	3.47	7.54	16.83	7.77	19.67	up
A_21_P0013035	LSM14A	6.38	5.36	0.49	5.09	0.41	down
A_33_P3338698	IHH	8.57	6.72	0.28	6.84	0.30	down
A_33_P3276282	PTPRF	4.58	3.40	0.44	3.46	0.46	down
A_23_P162171	MCAM	6.41	7.99	2.98	8.36	3.86	up
A_23_P110764	MYOT	5.12	2.44	0.16	2.44	0.16	down
A_32_P189211	C15orf56	5.67	1.95	0.08	1.89	0.07	down
A_23_P213699	NRG2	5.90	2.13	0.07	2.01	0.07	down
A_32_P55135	SSX1	4.22	5.24	2.03	5.48	2.40	up
A_24_P195081	CCDC171	2.30	4.66	5.13	4.11	3.49	up
A_21_P0010671	LYPLAL1-AS1	6.39	8.00	3.04	7.68	2.45	up
A_33_P3417517	BRD8	5.45	1.90	0.09	2.76	0.16	down
A_33_P3216605	TMEM63A	7.25	2.00	0.03	2.06	0.03	down
A_33_P3380502	GPR85	3.54	4.74	2.30	5.01	2.78	up
A_32_P360193	DNHD1	3.78	5.92	4.39	5.45	3.17	up

A_22_P00012044	C8orf37-AS1	5.69	4.68	0.50	4.21	0.36	down
A_24_P942068	TANC2	5.92	3.38	0.17	2.90	0.12	down
A_33_P3416797	OVOS2	4.66	3.38	0.41	2.55	0.23	down
A_33_P3379251	LRRC30	3.05	4.55	2.82	4.50	2.72	up
A_23_P169017	DEFB103B	2.31	4.72	5.30	6.84	23.03	up
A_24_P358805	CDHR1	4.74	2.29	0.18	1.82	0.13	down
A_33_P3798989	FLJ45950	5.12	3.41	0.31	3.89	0.43	down
A_23_P103703	HSPB7	3.21	4.24	2.05	4.82	3.05	up
A_33_P3239759	PPAN-P2RY11	6.02	4.74	0.41	4.56	0.36	down
A_23_P30649	SPACA1	5.47	3.12	0.20	3.78	0.31	down
A_23_P333640	PAPLN	9.45	8.38	0.48	8.17	0.41	down
A_33_P3331752	MUC3A	3.11	4.82	3.27	4.46	2.55	up
A_32_P45974	C2orf27A	4.53	2.05	0.18	3.35	0.44	down
A_23_P95851	TUBAL3	6.29	3.95	0.20	5.19	0.47	down
A_24_P148836	KLHDC8B	4.52	5.59	2.10	5.80	2.42	up
A_32_P138032	C1orf61	3.31	4.83	2.87	4.94	3.08	up
A_33_P3221443	ZNF254	4.16	5.22	2.09	5.25	2.12	up
A_33_P3233040	SERPINB11	5.84	4.48	0.39	4.75	0.47	down
A_23_P412515	CLDN12	4.86	6.32	2.76	6.18	2.51	up
A_23_P15182	ARL2BP	8.34	9.84	2.84	9.75	2.66	up
A_19_P00328832	AATBC	5.72	2.48	0.11	3.15	0.17	down
A_32_P70158	LILRB3	4.47	5.58	2.17	6.85	5.20	up
A_23_P41241	TGM4	5.35	2.02	0.10	2.00	0.10	down
A_24_P9883	AP5B1	7.25	5.78	0.36	5.83	0.37	down
A_33_P3379001	UNC5A	5.60	4.17	0.37	4.15	0.37	down
A_24_P267522	ZNF585A	3.43	4.74	2.47	5.14	3.26	up

A_23_P36531	TSPAN8	11.64	10.58	0.48	10.62	0.49	down
A_33_P3356462	C2CD4A	1.91	4.50	6.05	4.41	5.68	up
A_32_P69849	BEX5	3.60	5.39	3.48	5.13	2.89	up
A_23_P379475	DHCR24	11.35	9.95	0.38	10.27	0.47	down
A_33_P3294533	PRKCB	9.41	10.62	2.31	11.06	3.15	up
A_23_P104438	MYPN	5.56	3.98	0.34	4.09	0.36	down
A_23_P25030	HSD17B6	3.92	5.39	2.77	5.69	3.42	up
A_24_P354337	ARL6	3.62	5.40	3.45	5.51	3.71	up
A_33_P3292164	KRTAP9-7	3.36	5.10	3.36	5.63	4.84	up
A_24_P397386	LIFR	4.47	5.94	2.78	5.97	2.83	up
A_23_P11025	ZNF185	6.13	7.25	2.17	7.20	2.09	up
A_24_P305784	SPANXB1	8.35	9.83	2.79	10.24	3.71	up
A_33_P3396214	KREMEN2	2.72	5.24	5.73	4.70	3.92	up
A_24_P203000	IL2RB	3.69	5.55	3.64	5.45	3.39	up
A_24_P911094	RABL2A	3.40	4.95	2.92	4.85	2.73	up
A_22_P00014193	LHX5-AS1	6.68	3.19	0.09	2.26	0.05	down
A_23_P96285	REEP1	6.45	4.48	0.26	3.81	0.16	down
A_23_P370054	MAGEB18	6.38	1.79	0.04	3.30	0.12	down
A_33_P3394972	OSBPL5	4.40	3.35	0.48	3.09	0.40	down
A_33_P3266823	VWA5B2	5.36	2.03	0.10	3.73	0.32	down
A_23_P257834	ALB	10.11	9.05	0.48	8.32	0.29	down
A_23_P17065	CCL20	5.93	8.61	6.41	7.28	2.54	up
A_22_P00009881	ELFN2	4.52	2.12	0.19	3.08	0.37	down
A_23_P134340	NEUROD6	5.94	2.17	0.07	1.97	0.06	down
A_21_P0000876	TMEM161B-AS1	4.74	5.87	2.20	6.17	2.70	up
A_33_P3343010	HYPK	5.00	2.09	0.13	2.06	0.13	down

A_24_P291658	ADH1A	7.71	5.96	0.30	6.22	0.35	down
A_24_P102650	MUC5B	6.18	4.99	0.44	5.05	0.46	down
A_23_P258612	ATP8A2	5.47	6.66	2.28	6.65	2.25	up
A_33_P3402635	GAS8-AS1	5.77	1.87	0.07	3.03	0.15	down
A_23_P92161	ARL14	7.72	5.95	0.29	6.56	0.45	down
A_23_P141802	SERPINB7	3.72	4.89	2.25	5.49	3.40	up
A_24_P399980	HEPH	4.78	6.04	2.40	5.86	2.11	up
A_33_P3210223	ZCCHC6	5.05	3.73	0.40	3.65	0.38	down
A_23_P85453	CD244	5.08	2.14	0.13	2.06	0.12	down
A_24_P316601	C9orf47	1.74	4.73	7.99	4.19	5.46	up
A_32_P9382	MZT1	7.36	8.77	2.66	9.00	3.12	up
A_23_P400298	PRSS22	5.15	6.40	2.38	6.45	2.47	up
A_33_P3242909	ZNF563	6.45	3.14	0.10	3.78	0.16	down
A_24_P16856	AKAP17A	4.34	1.77	0.17	1.78	0.17	down
A_23_P58407	UGT2B15	6.03	2.06	0.06	3.67	0.19	down
A_33_P3290343	CYP1B1	11.11	9.80	0.40	9.87	0.42	down
A_21_P0000886	LDLRAD4-AS1	3.44	4.76	2.50	4.49	2.06	up
A_32_P66881	TLR4	8.50	7.29	0.43	7.44	0.48	down
A_33_P3413895	CDRT15P1	6.49	4.86	0.32	1.98	0.04	down
A_33_P3243093	RGS5	5.35	4.16	0.44	3.70	0.32	down
A_22_P00018514	NEURL1-AS1	5.06	1.89	0.11	1.89	0.11	down
A_33_P3327642	AIM1L	6.99	5.52	0.36	5.75	0.42	down
A_21_P0001706	PLEKHO1	3.75	4.78	2.03	4.80	2.07	up
A_24_P110983	AKT3	4.36	6.33	3.92	6.79	5.42	up
A_23_P311875	CD6	5.46	1.87	0.08	1.88	0.08	down
A_23_P436369	FILIP1	5.03	1.78	0.10	2.24	0.14	down

A_21_P0000437	SNORD115-2	6.41	1.86	0.04	1.86	0.04	down
A_33_P3233608	SPANXC	12.59	13.64	2.07	14.63	4.10	up
A_23_P16252	KLK1	6.28	7.47	2.28	7.45	2.26	up
A_23_P77908	SLC47A2	7.89	6.48	0.37	6.55	0.40	down
A_33_P3791118	ATP5L2	5.07	1.92	0.11	1.90	0.11	down
A_33_P3308862	NUTM2B	3.30	4.42	2.17	4.32	2.03	up
A_33_P3318002	PCDHA4	5.77	2.07	0.08	2.02	0.07	down
A_23_P99642	SLC7A7	10.52	9.40	0.46	9.37	0.45	down
A_33_P3219651	BMPER	5.27	2.30	0.13	2.35	0.13	down
A_23_P251499	PCOLCE	5.80	7.25	2.72	6.82	2.02	up
A_24_P106542	RSPO3	4.23	5.60	2.59	5.83	3.03	up
A_32_P52153	UNC5B-AS1	4.44	5.65	2.31	5.92	2.80	up
A_33_P3256317	MYT1L-AS1	4.49	1.72	0.15	1.72	0.15	down
A_23_P51761	OR6K2	4.28	6.71	5.38	6.46	4.53	up
A_33_P3251984	MRGPRF	6.19	4.82	0.39	4.90	0.41	down
A_23_P13294	OR1S2	4.57	2.19	0.19	2.68	0.27	down
A_33_P3682006	DBH-AS1	4.74	6.11	2.57	5.94	2.29	up
A_33_P3210399	SLC14A1	7.43	5.81	0.32	5.92	0.35	down
A_23_P16722	DOCK10	3.92	5.81	3.71	6.37	5.45	up
A_33_P3352382	ARG1	3.03	5.68	6.27	5.09	4.15	up
A_33_P3335825	PLCD4	5.51	2.88	0.16	1.85	0.08	down
A_33_P3405491	HCRP1	5.85	4.18	0.32	3.73	0.23	down
A_24_P48723	PTGIS	7.37	8.80	2.71	8.37	2.01	up
A_23_P429977	KCNQ1	4.61	5.96	2.55	6.38	3.39	up
A_23_P253536	NPR3	3.42	4.90	2.78	5.52	4.28	up
A_32_P125338	FAM43B	4.88	5.98	2.15	6.05	2.24	up

A_33_P3266550	ABO	4.80	3.26	0.34	3.79	0.49	down
A_23_P95790	ITLN1	6.51	5.46	0.48	4.52	0.25	down
A_33_P3419383	PDLIM3	3.57	5.06	2.81	4.79	2.33	up
A_24_P192805	CARD17	3.69	4.97	2.43	4.70	2.02	up
A_24_P316019	GOLGA8R	2.54	4.93	5.24	4.35	3.50	up
A_33_P3281741	RFX8	4.04	5.12	2.11	5.15	2.16	up
A_24_P402825	CACNA2D3	3.75	4.99	2.36	4.89	2.19	up
A_33_P3365621	OR2AT4	6.08	4.01	0.24	2.15	0.07	down
A_33_P3352467	SSTR2	6.14	4.78	0.39	1.91	0.05	down
A_32_P62963	KRT16P2	3.78	5.26	2.78	4.95	2.25	up
A_23_P338534	HIF3A	4.62	3.33	0.41	2.94	0.31	down
A_23_P18282	DLEC1	5.40	3.46	0.26	3.48	0.27	down
A_33_P3372389	NPHP4	3.14	4.37	2.35	4.23	2.13	up
A_23_P108082	CREB3L3	10.89	9.64	0.42	9.14	0.30	down
A_33_P3358943	GRM2	4.43	1.84	0.17	2.58	0.28	down
A_24_P11575	CRIM1	4.83	2.33	0.18	2.04	0.14	down
A_23_P338401	KDM1B	5.09	2.93	0.22	3.94	0.45	down
A_23_P122924	INHBA	7.07	8.17	2.14	8.27	2.29	up
A_23_P151436	ALG5	9.56	10.74	2.28	10.94	2.61	up
A_22_P00016162	BEAN1-AS1	6.42	1.99	0.05	2.64	0.07	down
A_33_P3422035	FXYD5	4.63	3.50	0.46	3.54	0.47	down
A_23_P23502	OR6N1	4.76	2.32	0.19	3.39	0.39	down
A_23_P91283	CASS4	5.15	3.39	0.30	2.89	0.21	down
A_23_P334955	FAM167A	4.08	5.40	2.50	5.56	2.78	up
A_23_P65472	RNASE11	4.92	1.83	0.12	1.83	0.12	down
A_23_P154500	DNMT3A	6.66	5.11	0.34	5.20	0.36	down

A_33_P3246163	RPL5	15.65	14.33	0.40	13.95	0.31	down
A_33_P3343175	CXCL10	5.59	4.31	0.41	3.88	0.31	down
A_23_P83599	PRKAR1B	6.41	7.49	2.11	7.41	2.00	up
A_22_P00009438	SVIL-AS1	5.10	4.03	0.48	4.01	0.47	down
A_21_P0004421	LUCAT1	9.18	10.27	2.13	10.51	2.52	up
A_23_P60166	DEPTOR	8.98	7.44	0.34	7.69	0.41	down
A_33_P3331437	ZNF518A	6.68	5.46	0.43	5.33	0.39	down
A_33_P3364741	MRC2	6.32	7.37	2.06	7.33	2.01	up
A_23_P328145	FAM71D	5.42	3.02	0.19	2.64	0.15	down
A_23_P4096	CA4	5.53	3.68	0.28	3.68	0.28	down
A_22_P00009585	BREA2	5.73	4.63	0.47	4.28	0.37	down
A_33_P3223338	PARVG	7.44	2.95	0.04	4.14	0.10	down
A_23_P82868	PLAT	6.62	8.61	3.97	8.10	2.78	up
A_21_P0013259	GS1-259H13.2	4.10	5.38	2.43	5.23	2.19	up
A_23_P81158	ADH1C	11.84	10.17	0.31	10.01	0.28	down
A_23_P40096	PROC	5.37	6.77	2.65	6.38	2.02	up

Supplementary Table S5. KEGG pathway enrichment of DEGs

Pathways	P value	Gene list
TNF signaling pathway	0.0007	CSF2, IL18R1, IL6, PTGS2, CCL20, CSF1, PIK3CD, CREB3L3, AKT3, CXCL10
Complement and coagulation cascades	0.0010	PLAT, KNG1, MBL2, F5, C3, CFI, C1QC, PROC
cAMP signaling pathway	0.0058	HTR1B, SSTR2, PTGER3, HTR1A, NPY, PIK3CD, CAMK2B, GRIN3B, CREB3L3, GHSR, AKT3, LIPE
HIF-1 signaling pathway	0.0072	IL6, TFRC, PIK3CD, ENO2, TLR4, CAMK2B, AKT3, PRKCB
Platelet activation	0.0100	FYN, PIK3CD, GUCY1A2, TBXA2R, COL1A1, APBB1IP, COL5A2, PLA2G4E, AKT3
Retinol metabolism	0.0165	CYP2B6, ADH1C, HSD17B6, ADH1A, UGT2B15, RETSAT
Cytokine-cytokine receptor interaction	0.0167	CSF2, IL18R1, IL2RB, IL6, PDGFB, CCL20, CSF1, CRLF2, LIFR, CXCL12, CSF2RA, CXCL10
Fc epsilon RI signaling pathway	0.0197	CSF2, FYN, PIK3CD, PLA2G4E, AKT3, PRKCB
Synthesis and degradation of ketone bodies	0.0210	HMGCLL1, HMGCS2, HMGCL
Butanoate metabolism	0.0233	GAD2, HMGCLL1, HMGCS2, HMGCL
Metabolism of xenobiotics by cytochrome P450	0.0273	CYP1B1, CYP2B6, ADH1C, ADH1A, UGT2B15, GSTM5
Chemical carcinogenesis	0.0366	CYP1B1, PTGS2, ADH1C, ADH1A, UGT2B15, GSTM5
Insulin resistance	0.0378	IL6, CD36, PTPRF, PIK3CD, CREB3L3, SLC27A2, AKT3
Proteoglycans in cancer	0.0408	WNT10A, PIK3CD, HSPG2, TLR4, CAMK2B, DCN, MMP2, AKT3, PRKCB, TWIST1
Cholinergic synapse	0.0424	FYN, PIK3CD, CAMK2B, CREB3L3, KCNQ1, AKT3, PRKCB
Hematopoietic cell lineage	0.0456	CSF2, IL6, CD36, TFRC, CSF1, CSF2RA
Tyrosine metabolism	0.0457	TYR, ADH1C, ADH1A, HPD
Jak-STAT signaling pathway	0.0494	CSF2, IL2RB, IL6, CRLF2, PIK3CD, LIFR, AKT3, CSF2RA
ErbB signaling pathway	0.0496	PIK3CD, CAMK2B, NRG1, NRG2, AKT3, PRKCB
