# Supplementary figures and images for: UGT2B15 Acts as a Critical Detoxification Barrier Against Chemi-Cal-Induced Hepatotoxicity and Carcinogenesis via the Androgen Receptor Axis
Source: Cells. 2026 Apr 30;15(9):824. doi: 10.3390/cells15090824 (PMC13162611; doi:10.3390/cells15090824)

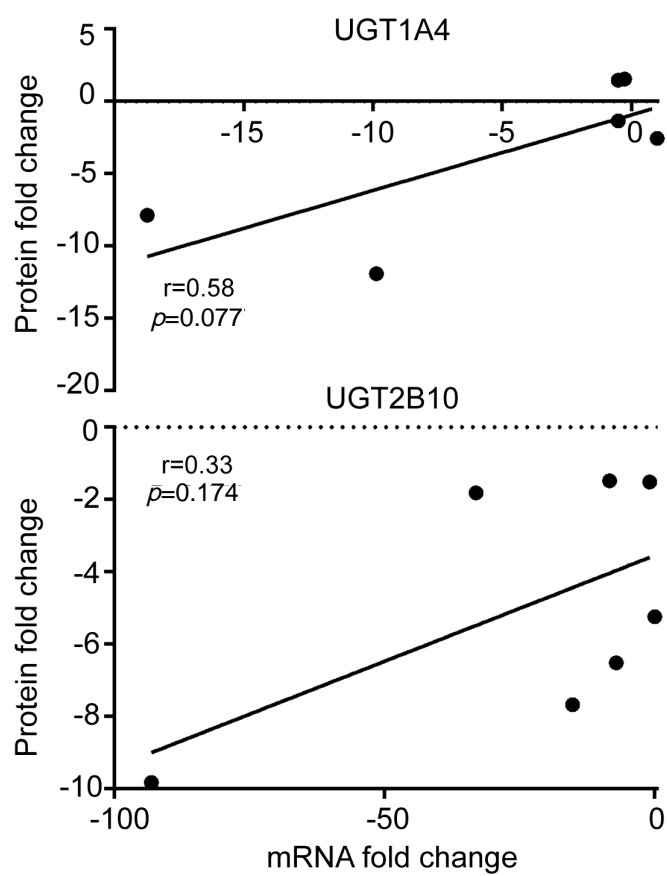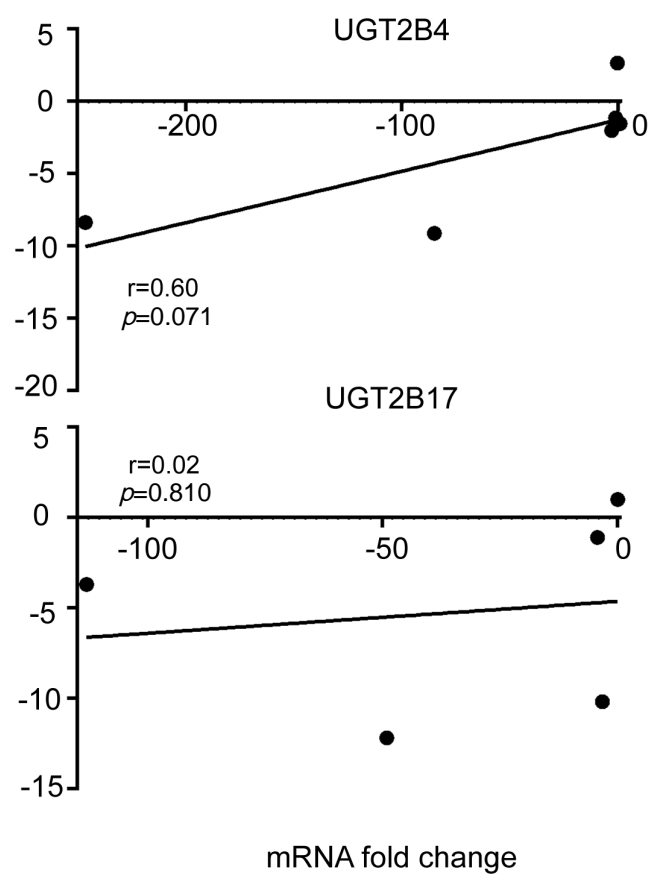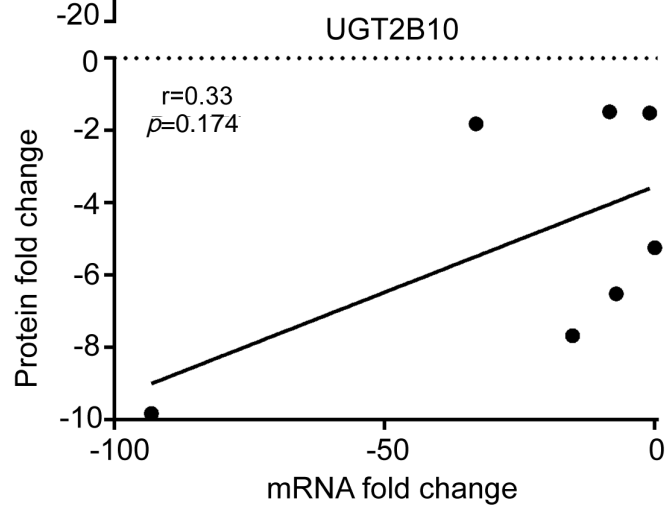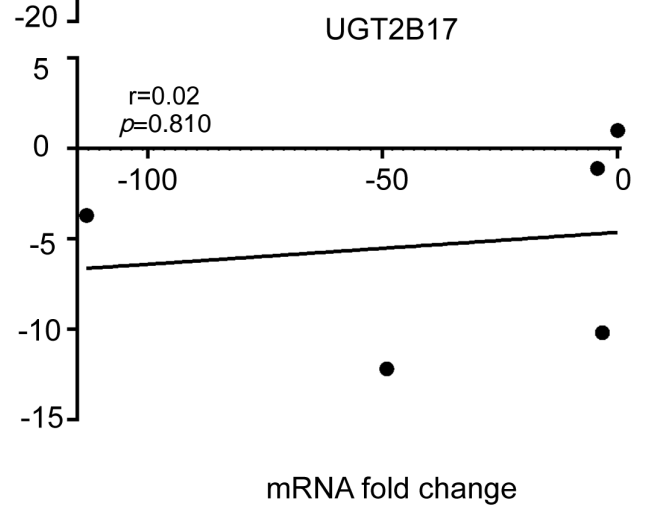

Supplement: Supplementary file 1 [file cells-15-00824-s001.zip › Figure S1._FIN..pdf]

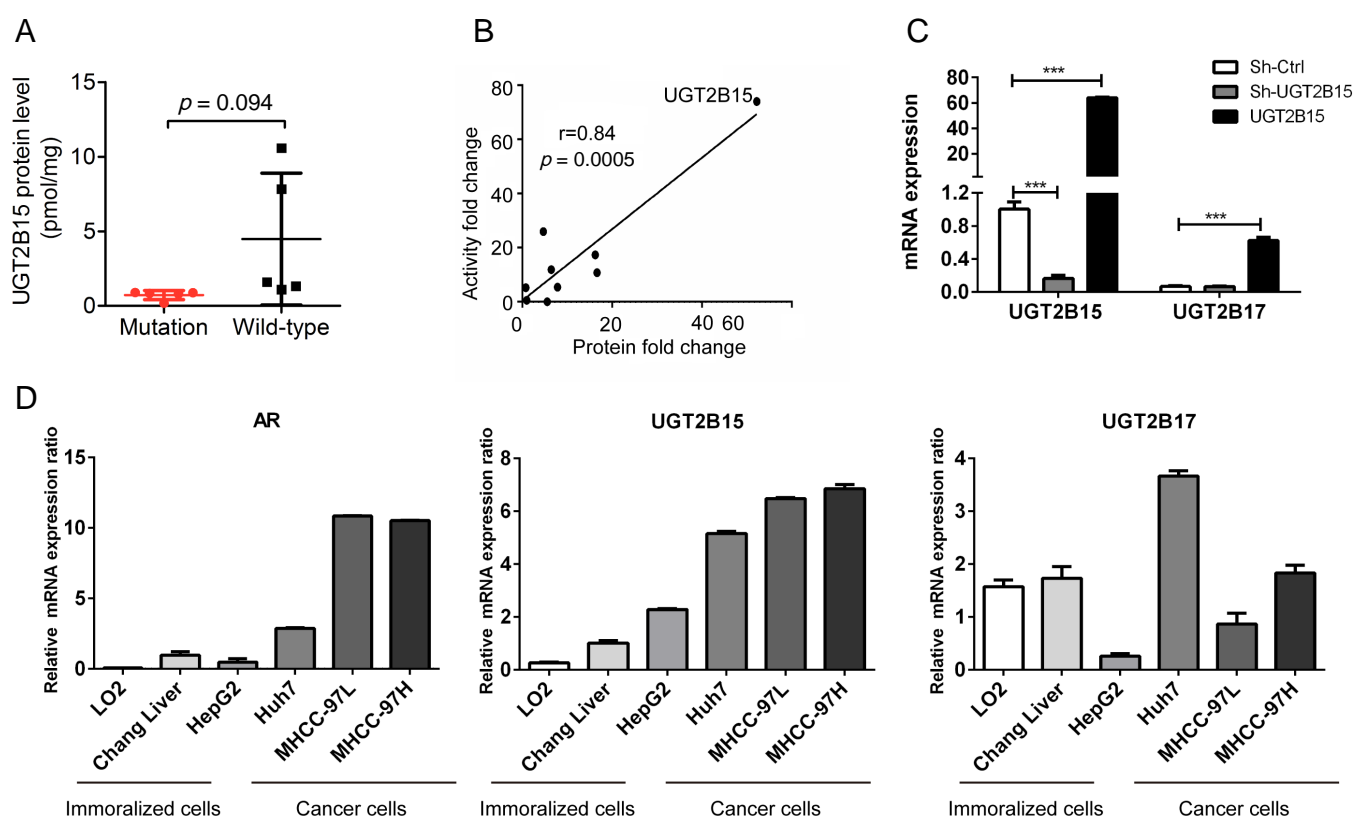

Supplement: Supplementary file 1 [file cells-15-00824-s001.zip › Figure S2._FIN..pdf]

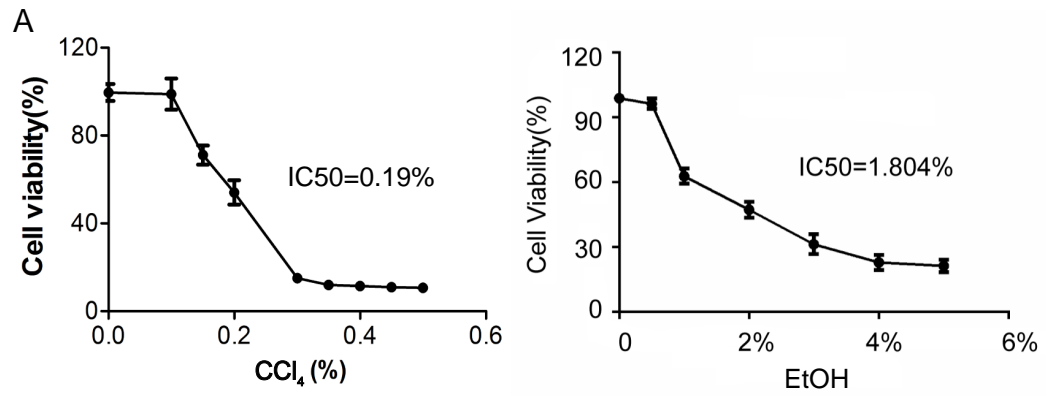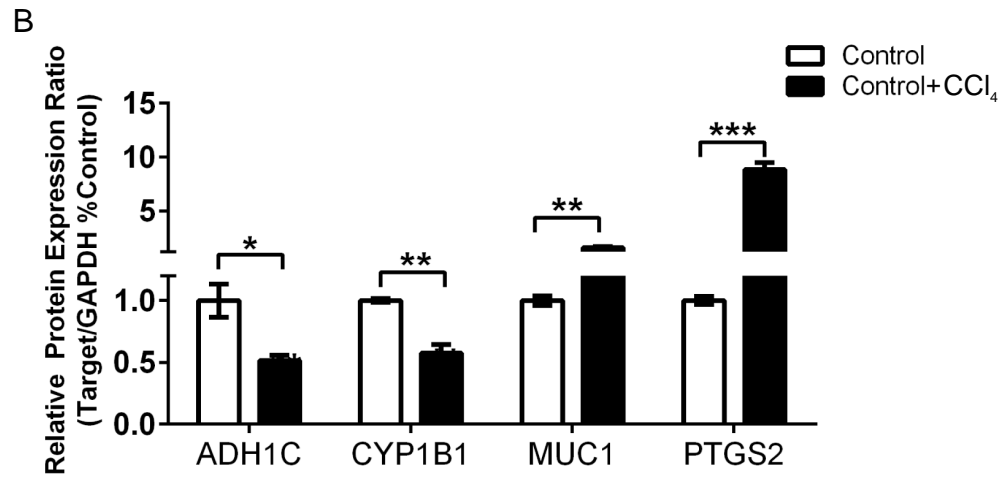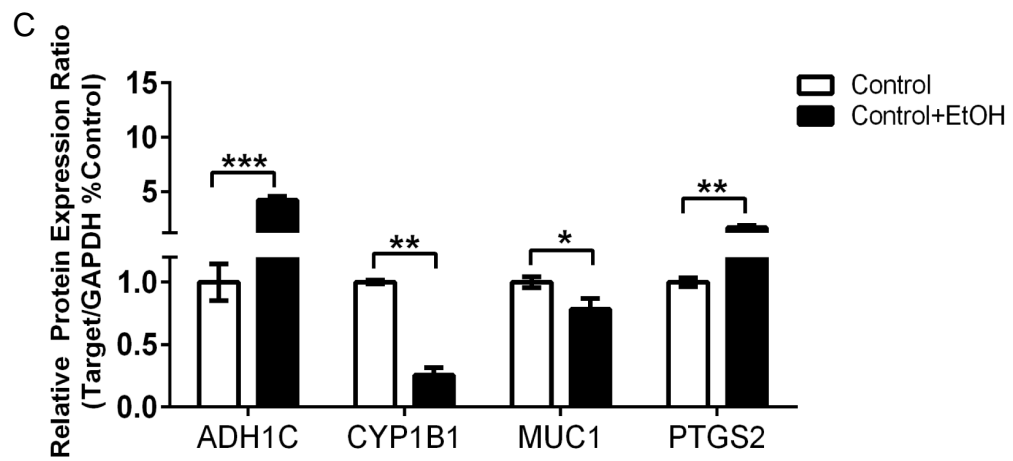

Supplement: Supplementary file 1 [file cells-15-00824-s001.zip › Figure S3._FIN..pdf]
